# An N-terminal conserved region in human Atg3 couples membrane curvature sensitivity to conjugase activity during autophagy

**DOI:** 10.1038/s41467-020-20607-0

**Published:** 2021-01-14

**Authors:** Yansheng Ye, Erin R. Tyndall, Van Bui, Zhenyuan Tang, Yan Shen, Xuejun Jiang, John M. Flanagan, Hong-Gang Wang, Fang Tian

**Affiliations:** 1 Departments of Biochemistry and Molecular Biology, Penn State College of Medicine, Hershey, PA USA; 2 Department of Pediatrics, Penn State College of Medicine, Hershey, PA USA; 3 Laboratory of Chemical Physics, National Institute of Diabetes and Digestive and Kidney Diseases, US National Institutes of Health, Bethesda, MD USA; 4 Cell Biology Program, Memorial Sloan Kettering Cancer Center, New York, NY USA

**Keywords:** Enzymes, Solution-state NMR, Biophysics

## Abstract

During autophagy the enzyme Atg3 catalyzes the covalent conjugation of LC3 to the amino group of phosphatidylethanolamine (PE) lipids, which is one of the key steps in autophagosome formation. Here, we have demonstrated that an N-terminal conserved region of human Atg3 (hAtg3) communicates information from the N-terminal membrane curvature-sensitive amphipathic helix (AH), which presumably targets the enzyme to the tip of phagophore, to the C-terminally located catalytic core for LC3–PE conjugation. Mutations in the putative communication region greatly reduce or abolish the ability of hAtg3 to catalyze this conjugation in vitro and in vivo, and alter the membrane-bound conformation of the wild-type protein, as reported by NMR. Collectively, our results demonstrate that the N-terminal conserved region of hAtg3 works in concert with its geometry-selective AH to promote LC3–PE conjugation only on the target membrane, and substantiate the concept that highly curved membranes drive spatial regulation of the autophagosome biogenesis during autophagy.

## Abstract (web summary)

The E2-like enzyme human Atg3 catalyses the transfer of ubiquitin-like mammalian LC3 to the lipid phosphatidylethanolamine during autophagosome formation. Here, the authors combine NMR measurements with in vitro biochemical and in vivo cellular assays and show that the N-terminal conserved region of human Atg3 communicates information from the curvature-sensing domain to its active site.

## Introduction

Macroautophagy (autophagy) is a highly conserved stress response in eukaryotes^1–3^. The process begins with the assembly of the phagophore (a cup-shaped vesicle), which, in turn, undergoes membrane expansion via a largely unknown mechanism and eventually seals to form the autophagosome (a double-membrane organelle). Formation of this organelle results in encapsulation of its cargo for degradation following fusion with the lysosome. The entire process requires the coordination of more than 30 autophagy-related (Atg) proteins in a spatially and temporally controlled manner^4–6^. The hallmark of the autophagosomal membrane is the covalent attachment of LC3, one of the homologs of yeast Atg8, directly to the amino group of phosphatidylethanolamine (PE) lipids in the membrane resulting in the LC3–PE complex (also referred as LC3-II)^7^. The conjugation of LC3 to PE lipids proceeds in three sequential steps: first, the cysteine protease Atg4 removes the last (or a few) C-terminal residue of LC3; second, Atg7, an E1-like activating enzyme, activates LC3 and catalyzes the formation of a thioester intermediate (LC3-Atg3) with an E2-like enzyme Atg3; third, the Atg5–Atg12/Atg16 complex, a presumed E3-like enzyme, promotes the transfer of LC3 from LC3-Atg3 to PE. The formation of LC3–PE triggers phagophore expansion and acts as an adaptor for sequestering cargos for breakdown^7,8^.

The structures of yeast and *Arabidopsis thaliana* Atg3 homologs reveal a catalytic core with structural and presumably mechanistic similarity to members of the E2 ligase family, despite having little sequence similarity^2,9–11^. In vivo, Atg3 functions in concert with the Atg12–Atg5/Atg16 complex, which is thought to allosterically activate Atg3 for conjugation of LC3 to PE in membranes^12,13^. However, in vitro, Atg3 alone can catalyze the conjugation, although the addition of the Atg12–Atg5/Atg16 complex increases activity. Targeting Atg3 to the membranes containing PE substrates requires its N-terminus, which seemingly is not part of the more C-terminally located catalytic core. Removal of the first 3 residues of yeast Atg3 (yAtg3) results in an enzyme with reduced activity, and deletion of the first 7 residues abolishes Atg8–PE conjugation^14^. It has been suggested that residues 3–13 form an amphipathic helix (AH) that targets the enzyme to the membrane, since disrupting the hydrophobic face, or swapping the equatorial charged residues, severely reduces Atg8–PE conjugation in vivo and in vitro^14^. Furthermore, the corresponding putative AH region of mouse Atg3 (mAtg3) has recently been shown to be responsible for its membrane curvature-dependent conjugation activity; the catalytic activity of mAtg3 for LC3–PE formation markedly increases as the size of liposomes decreases below 50 nm^15,16^. This has led to the suggestion that the N-terminal AH guides the enzyme to the leading edge of an expanding phagophore, where the membrane is highly curved with a radius possibly as small as 10 nm^17^. Here, we demonstrate that the N-terminus is not merely a membrane curvature-selective anchor; membrane binding, while necessary, is not sufficient for catalyzing LC3–PE conjugation.

**Fig. 1:**
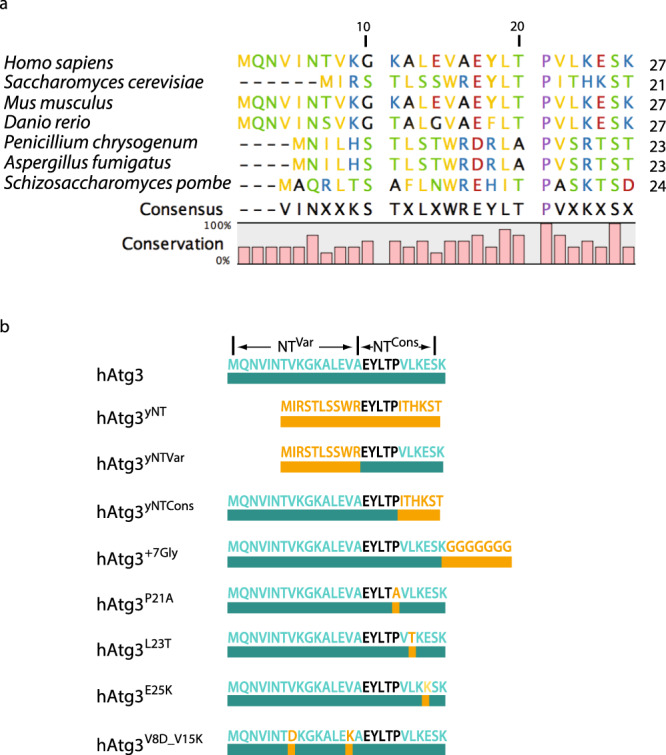
The N-terminals (NTs) of Atg3 homology have variable and conserved regions. **a** Sequence analysis of Atg3 from multiple species, as well as the consensus sequence. All sequences begin at the first residue of the protein; residues numbered at the top refer to the human sequence. Non-polar and aromatic residues are in yellow, polar residues in green, positively charged and histidine residues in blue, negatively charged residues in red, alanine and glycine residues in black, and proline residue in purple. **b** Diagrams of hAtg3 NT constructs prepared in this study. Sequences corresponding to the hAtg3 sequence are teal and mutations and insertions are gold.

Sequence analysis of the Atg3 N-terminus from multiple species reveals a highly conserved region extending from residues 17 to 26 of hAtg3 (Fig. 1a). This region includes the last three resides of the N-terminal AH (residues 3–19), and the corresponding region in the yeast and *A. thaliana* homologs is not observed in existing crystal structures. We discovered that this conserved region plays a previously undescribed role in linking the membrane curvature-sensing role of the AH to its PE conjugase activity. Using LC3B, a mammalian homolog of yeast Atg8, we show that mutations in this region greatly reduce or abolish LC3B–PE conjugations in the in vitro and in vivo assays, despite retaining normal liposome binding and formation of the thioester LC3B-hAtg3 intermediate. NMR chemical shift perturbation (CSP) experiments indicate that structural rearrangements in the C-terminal part of hAtg3 (including the active site) occur upon the binding of its N-terminal AH to the membrane, and that the conserved region is critical for inducing these functionally relevant structural changes. Together, our results support the hypothesis that tight coordination of the membrane curvature-sensitive interaction of hAtg3 with its LC3–PE catalytic activity helps ensure that the biogenesis of the autophagosome proceeds in a spatially regulated manner.

## Results

### Sequence analysis reveals conserved and variable regions in Atg3’s N-terminus

Figure 1a displays the sequence alignment of the 27 residues of the N-terminal of hAtg3 (herein referred to as NT), which are unstructured in aqueous solution, to the analogous region in selected organisms. Despite the fact that the NT is essential for protein activity, this region sharply diverges in sequence identity and length, with fungi tending to have shorter helices and more large hydrophobic residues compared to other kingdoms (however, all animal forms share high homology, Supplementary Fig. 1). The observed sequence alignments also suggest that the NT region can be divided into two sub-regions (Fig. 1a): a variable region (residues 1–16 of hAtg3, NT^Var^) and a conserved region (residues 17–26 of hAtg3, NT^Cons^) which includes a highly conserved sequence motif (EYLTP). These differences are best illustrated by comparing the NTs of *Homo sapiens* and *Saccharomyces cerevisiae* Atg3. The yeast variable region is shorter than that seen in humans and contains a more hydrophobic set of residues, while the conserved region shares noticeable homology with the human Atg3. The NT^Var^ region makes up most of hAtg3 AH (residues 3–19, defined by our NMR study described below) which is responsible for the protein’s membrane curvature-dependent binding, but a functional role of the NT^Cons^ has not yet been reported. In the following studies, we examine the roles of NT^Cons^ in membrane binding, formation of the thioester LC3B-hAtg3 intermediate, and LC3B–PE conjugation, using established in vitro and in vivo assays^17^ and NMR.

**Fig. 2:**
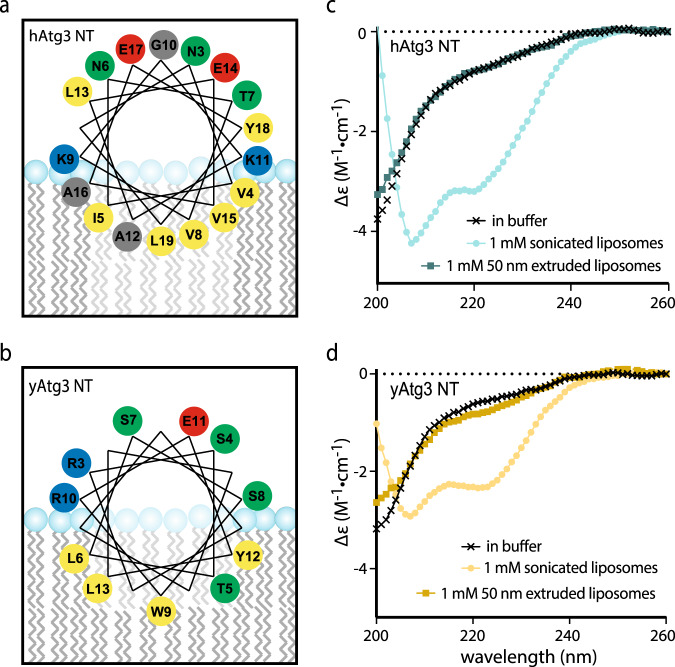
Atg3 NTs have conserved membrane curvature sensitivity. **a**, **b** Helical wheel plots for hAtg3 and yAtg3 amphipathic helix. Residues are colored as in Fig. 1a. **c**, **d** CD spectra of hAtg3 NT and yAtg3 NT, respectively, in the absence and presence of 10% DOPG, 90% POPC sonicated, and 50 nm extruded liposomes. Average sizes for sonicated and 50 nm extruded liposomes, measured by dynamic light scattering, are 32 and 56 nm, respectively.

We first determined whether the NT^Var^ of yAtg3 has membrane curvature-sensing properties, since it shares poor sequence similarity with hAtg3’s NT^Var^. Helical wheel plots for both NTs up to the conserved Leu19 display quite different chemical characteristics (Fig. 2a, b). The polar face of the hAtg3 AH contains multiple charges including two equatorial lysines, while its non-polar face is rich in hydrophobic amino acids with small side chains such as valine and alanine. However, the polar face of the yAtg3 AH is rich in serines and threonines and its non-polar face has large hydrophobic residues such as tryptophan and leucine. This pattern of residues is a characteristic feature of an ALPS (amphipathic lipid packing sensor) motif^18^. To determine whether both AHs demonstrate similar membrane curvature-sensing behavior, we synthesized two peptides that correspond to the sequences of the NT regions of hAtg3 and yAtg3 (Fig. 1a) and examined their secondary structure in the presence and absence of lipid vesicles of different sizes using circular dichroism (CD) spectroscopy. Both peptides showed the spectral features of a random coil in aqueous solution (Fig. 2c, d). This is consistent with the behavior of typical AH peptides. In the presence of sonicated liposomes (average sizes of 32 nm measured by dynamic light scattering) but not 50 nm extruded liposomes (average sizes of 56 nm measured by dynamic light scattering), the CD spectra of both were more representative of that expected for α-helical peptides. Furthermore, the observed coil to helix transition requires the presence of negatively charged lipids in liposomes^16^ but is not sensitive to exact lipid composition and PE is not essential (Supplementary Fig. 2). This indicates that the membrane curvature sensitivity of Atg3’s AH is conserved among homologs, likely through different curvature-sensing mechanisms because of their lack of sequence conservation^18,19^.

### The NT^Cons^ of hAtg3’s NT is key to its activity

**Fig. 3:**
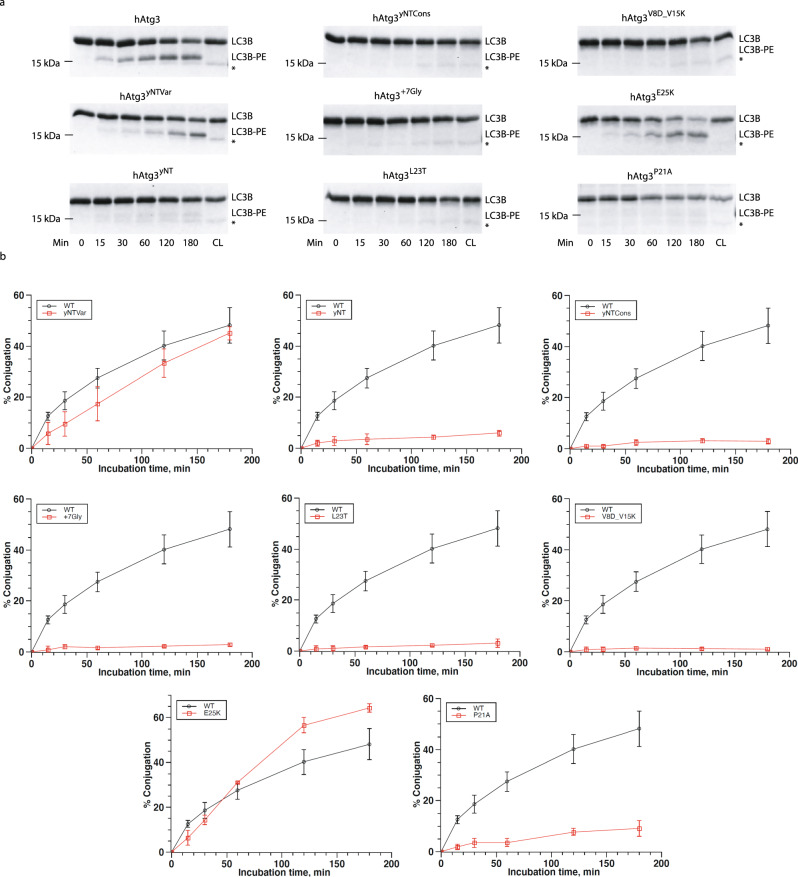
The N-terminal conserved region of hAtg3 is critical for protein function. **a** SDS-PAGE gel images of time-dependent formation of LC3B–PE for hAtg3, hAtg3^yNTVar^, hAtg3^yNT^, hAtg3^yNTCons^, hAtg^+7Gly^, and for hAtg3^P21A^, hAtg3^L23T^, hAtg3^E25K^ as well as hAtg3^V8D_V15K^. Gels were quantified with ImageJ; conjugation percentage of LC3B–PE was determined by the amount of LC3B–PE divided by the total LC3B present. CL is the control without liposomes. * Asterisk indicates a small amount of degradation of LC3B in the presence of ATP. **b** Plots of time-dependent formation of LC3B–PE for hAtg3 and mutants. Data are presented as mean ± SD. Quantification of conjugation reactions were obtained from three separate measurements (*n* = 3).

Capitalizing on the fact that the AH region of NTs, as a whole, shows conserved membrane curvature sensitivity, we prepared three chimeric constructs to investigate the functional significance of the hAtg3’s NT^Cons^ region: hAtg3^yNTVar^, hAtg3^yNTCons^, and hAtg3^yNT^ (replacing the NT’s variable region, conserved region, and the entire NT of hAtg3 with that of yAtg3, respectively, Fig. 1b). In the in vitro conjugation assay, hAtg3^yNTvar^ retained about 90% of the wild-type activity (Fig. 3a and Supplementary Fig. 3a), suggesting that the curvature-sensing region of yAtg3’s NT is capable of directing the chimeric proteins to the targeted membrane, as expected from our peptide study. Surprisingly, hAtg3^yNTCons^ and hAtg3^yNT^ effectively abolish LC3B–PE conjugation (Fig. 3 and Supplementary Fig. 3a). In order to investigate whether the spatial arrangement of the NT^Cons^ region to the rest of protein is important, we designed a mutant with a linker of seven Gly residues inserted between residues Lys27 and Phe28 (hAtg3^+7Gly^). We reasoned that this flexible linker presumably would “decouple” the membrane binding activity of hAtg3 AH from its conjugase activity. In the in vitro conjugation assay hAtg3^+7Gly^ shows little activity (Fig. 3 and Supplementary Fig. 3a). These results suggest that the NT^Cons^ region plays a critical role in enzyme activity.

To examine whether specific residues in the NT^Cons^ region are important for LC3B–PE conjugation, we constructed three variant forms: hAtg3^L23T^, hAtg3^E25K^, and hAtg3^P21A^ (Fig. 1b). The first two (hAtg3^L23T^ and hAtg3^E25K^) substitute residues that differ most in their side chain properties between the human and yeast NT^Cons^ regions and may account for the inability of the hAtg3^yNTCons^ and hAtg3^yNT^ to support LC3B–PE conjugation. The third substitution (hAtg3^P21A^) replaces an invariant proline residue with an alanine. It was chosen because proline has been shown to perform a critical role in membrane curvature-sensing AHs^20^. In the in vitro conjugation assay, hAtg3^E25K^ produces about 30% more LC3B–PE than the wild-type protein does after 2 h, but hAtg3^L23T^ and hAtg3^P21A^ have severely reduced conjugation activity in vitro (Fig. 3 and Supplementary Fig. 3a). The results of CD and liposome co-sedimentation assays^21,22^ allow us to rule out the possibility that a defect in protein–membrane interactions is a major contributor to the effects of these substitutions. All mutants interact with the liposomes in an analogous manner to wild-type protein except that the binding of hAtg3^L23T^ is reduced by about 40% (Supplementary Figs. 4a, b and 5). As a control, we also examined the AH folding and liposome binding of hAtg3^V8D_V15K^, a mutant designed not to interact with the membrane^15^. As expected, the NT^V8D_V15K^ peptide is unfolded in the presence of liposomes and the hAtg3^V8D_V15K^ protein shows minimal levels of liposome association (Supplementary Figs. 4c and 5), which is similar to an hAtg3 construct without the first 25 residues. Furthermore, all variants can form a normal covalent thioester intermediate with LC3B (Supplementary Fig. 3b), indicating that these mutations do not affect their interactions with the E1-like Atg7. Together, these results indicate that the binding of AH to the membrane is necessary but not sufficient to promote the LC3B–PE conjugation, and functional forms of both the AH and the NT^Cons^ region of hAtg3 are required in vitro.

### hAtg3^P21A^ and hAtg3^L23T^ mutants do not restore LC3B lipidation and autophagic flux in Atg3 knockout cells

**Fig. 4:**
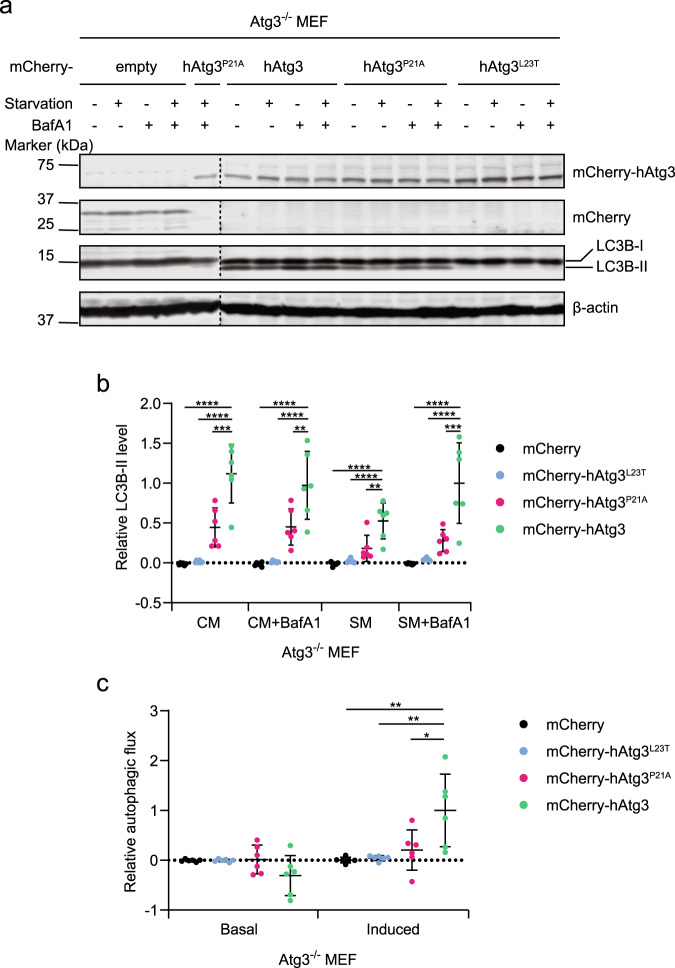
hAtg3^P21A^ and hAtg3^L23T^ are impaired in inducing LC3B lipidation and autophagic flux in vivo. Atg3 knockout (Atg3^−/−^) mouse embryonic fibroblasts (MEFs) stably expressing mCherry, mCherry-hAtg3, mCherry-hAtg3^P21A^, or mCherry-hAtg3^L23T^ were cultured in complete media (CM) and starvation media (SM) with or without bafilomycin A1 (BafA1) for 3 h and subjected to immunoblotting with the indicated antibodies. **a** Representative immunoblot (*n* = 6 blots). **b** Quantitative analysis of the relative LC3B–II level (*n* = 6 blots). **c** Relative basal and starvation-induced autophagic flux (*n* = 6 blots). Statistical analysis was performed using one-way ANOVA test followed by Dunnett’s multiple comparisons test. Data are presented as mean ± SD. *P* values: ^****^P < 0.0001 (**b**), ^***^P = 0.0001 and 0.0003 (left to right in **b**), ^**^P = 0.0037 and 0.0011 (left to right in **b**) and 0.0014 and 0.0021 (left to right in **c**), and ^*^P = 0.0097 (**c**).

To determine whether the NT^Cons^ region is essential for LC3B–PE conjugation and autophagic flux in the presence of additional autophagy machinery in mammalian cells, we reintroduced wild-type hAtg3, hAtg3^L23T^, or hAtg3^P21A^ variant into Atg3^−/−^ mouse embryonic fibroblasts (MEFs) by lentiviral transduction and examined LC3B lipidation and autophagic flux by monitoring the lysosomal turnover of LC3B-II in the presence or absence of the lysosomal inhibitor bafilomycin A1 (BafA1), as described previously^23^. While the lipidation of LC3B was absent in Atg3^−/−^ MEFs, the expression of hAtg3 rescued not only LC3B–PE conjugation, but also starvation-induced autophagic flux (Fig. 4a–c). In contrast, the introduction of hAtg3^L23T^ into Atg3^−/−^ MEFs nearly completely failed to induce LC3B-II production and autophagic flux, whereas the introduction of hAtg3^P21A^ partially rescued LC3B lipidation (Fig. 4a, b). The LC3B-II level of Atg3^−/−^ cells expressing hAtg^P21A^ was significantly lower compared to that of Atg3^−/−^ cells expressing hAtg3. Although there was no statistical difference in the basal autophagic flux, nutrient starvation induced a significantly high level of LC3B-II lysosomal turnover in Atg3^−/−^ MEFs expressing hAtg3 compared to the empty vector, hAtg3^L23T^ or hAtg3^P21A^ (Fig. 4c). Collectively, the data strongly support that the NT^Cons^ region of Atg3 is important for LC3B lipidation and autophagic flux in vivo.

### NMR chemical shift perturbations provide molecular insights into the functional role of hAtg3’s NT conserved region

Our in vitro biochemical and in vivo cellular experiments demonstrate that the hAtg3 NT^Cons^ region is required for LC3B–PE conjugation. To discern the molecular basis of this regulation, we performed solution NMR CSP experiments. hAtg3 is a dynamic protein and more than 1/3 of its 314 residues are expected to be in unstructured regions, based on existing Atg3 structures. To facilitate NMR studies, we first optimized hAtg3 constructs. Deletion of the unstructured region from residues 90 to 190 (hAtg3^∆90-190^) visibly improves NMR spectral quality (Supplementary Fig. 6). Moreover, hAtg3^∆90-190^ retains about 50% of its conjugase activity compared to the wild type (Supplementary Fig. 7). The reduced conjugation activity of hAtg3^∆90-190^ is at least partially due to a slow formation of the LC3B-hAtg3^∆90-190^ intermediate as a result of removing its major Atg7 interacting site (residues 157–181)^11,24,25^. As shown in Supplementary Fig. 8a, LC3B-hAtg3 intermediate formation for the wild-type hAtg3 is near complete (~70%) within 30 min and only about ~25% of the intermediate is formed for the hAtg3^∆90-190^ construct after 1 h, despite the fact that the stability of LC3B-hAtg3 and LC3B-hAtg3^∆90-190^ intermediates in aqueous solution is comparable (Supplementary Fig. 8b). Additionally, the LC3B–PE formation for the hAtg3^∆90-^^190^, but not hAtg3, can be improved by increasing the concentration of mAtg7 (mouse Atg7) in the reaction buffer (Supplementary Fig. 8c). When recapitulating the NT point mutations in this 90–190 deletion construct, the hAtg3^∆90-190_P21A^ shows decreased activity when compared to wild-type protein and the hAtg3^∆90-190_L23T^ has nearly abolished conjugation activity, consistent with the behavior of the full-length proteins (Supplementary Fig. 8d).

Using TROSY-based assignment experiments, we assigned about 95% of its backbone resonances (Supplementary Fig. 9). The majority of unassigned residues (residues 260–269) are near the active site C264 and their resonances are not observed, most likely as a result of intermediate exchange. This indicates that the active site region of hAtg3 is probably involved in millisecond motions in aqueous solution. Interestingly, similar dynamics have been observed for yAtg3 in a recent study^26^. In addition, we prepared an hAtg3 construct without the 25 N-terminal residues (hAtg3^∆1-25^, Supplementary Fig. 10) and assigned about 80% of its backbone and C_β_ resonances despite the fact that this construct contains a long stretch of ~100 unstructured residues (to be published). Using assigned shifts of ^13^C_α_, ^13^C_β_, ^13^C’, ^15^N, and ^1^H^N^, we generated structural models for hAtg3^∆1-25^ with the PONOMA program^27^. Out of 2000 calculated models, the ten lowest-energy predictions show good convergence with an RMSD of 1.7 Å for structured regions (Supplementary Fig. 11).

In order to identify a membrane model that is compatible with high-resolution NMR studies of hAtg3 and supports its conjugase activity, we tested whether several commonly used membrane mimics including micelles, nanodiscs, and bicelles could replace liposomes in the in vitro conjugation assay. Consistent with a previous report^28^, LC3B–PE conjugation does not occur in LPC/LPG/LPE micelles (Supplementary Fig. 12a). In addition, no LC3B–PE conjugation is detected in POPC/POPG/POPE nanodiscs (Supplementary Fig. 12b). This is most likely due to the lack of strong interactions between hAtg3 NTs and the surface of the relatively tightly packed lipid bilayers. Consistent with this, only small CSPs and intensity perturbations (characteristics of transient interactions) are observed in the TROSY spectra of hAtg3^∆90-190^ with and without nanodiscs (Supplementary Fig. 13). By comparison, the LC3B–PE conjugation reaction in DMPC/DMPG/LPE/DHPC bicelles proceeds at a rate comparable to that in liposomes (Supplementary Fig. 12c). The dynamic planar surfaces of bicelles are loosely packed and presumably mimic a membrane with the type of packing defects that are required for interactions with hAtg3. The observation that hAtg3 is more active in larger bicelles (Supplementary Fig. 12c), where the planar faces have a proportionally larger fraction of the bicelle’s surface, further supports the notion that the protein mainly interacts with the planar faces instead of the highly curved edges of bicelles. We also examined the LC3B–PE conjugation reaction in bicelles for the NT point mutants. As shown in Supplementary Fig. 12c, the hAtg3^P21A^ and hAtg3^V8D_V15K^ constructs shows much reduced or no activity, consistent with the results from in vivo cellular assay and in vitro conjugation assay using liposomes. However, in bicelles the hAtg3^L23T^ construct functions differently. In contrast to its almost total inactivity in the in vitro conjugation assay when using liposomes and the in vivo cellular assay, the hAtg3^L23T^ construct shows moderate conjugation activity using bicelles. As a result of these observations, we have focused the NMR structural analysis in bicelles on the hAtg3^∆90-190^ and hAtg3^∆90-190_P21A^ constructs.

**Fig. 5:**
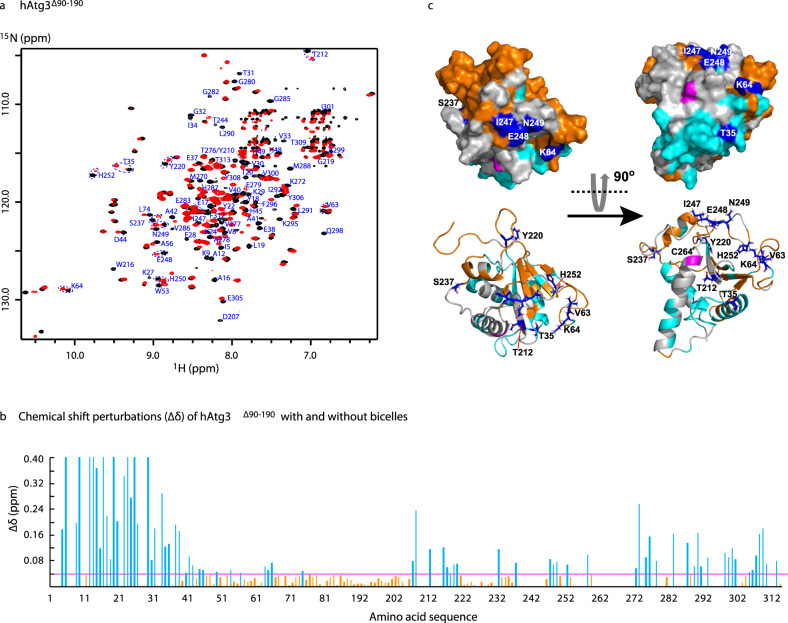
Binding of hAtg3^∆90-190^ to bicelles results in structural arrangements beyond its NT. **a** Overlay of ^2^H,^15^N,^13^C-labeled hAtg3^∆90-190^ TROSY spectra acquired on a Bruker 600 MHz spectrometer at 25 °C, pH 7.5 in the absence (black) and presence (red) of bicelles (DMPC:DMPG:DHPC = 4:1:20, molar ratio). **b** Plots of hAtg3^∆90-190 15^N and ^1^H chemical shift perturbations (CSPs, ∆δ) upon interaction with bicelles against residue numbers. ${{\Delta }}\delta = \sqrt {0.5^\ast \{ {( {\frac{{\delta _{\mathrm{N}}}}{5}} )^2 + \delta _{\mathrm{H}}^2} \}}$, $\delta _{\mathrm{H}}$ and $\delta _{\mathrm{N}}$represent the changes in ^1^H and ^15^N chemical shifts upon interacting with bicelles, respectively. **c** Surface and ribbon representations of a hAtg3 structural model with perturbed residues (∆δ ≥0.04 ppm) colored cyan and unperturbed residues (∆δ < 0.04 ppm) colored orange. The active site C264 is highlighted in magenta and unassigned residues are colored white. Some resonances displaying large CSPs are circled in (**a**) and their corresponding residues are colored blue in (**c**).

Figure 5a shows an overlay of the TROSY spectra of hAtg3^∆90-190^ in the absence (black) and presence (red) of bicelles, in which pronounced spectral changes are observed. Despite the large size of bicelles and exchange broadening, we have been able to assign about 80% of the hAtg3 residues in the bicelle-bound state. Perturbed resonances (some of which are labeled with assignments in the spectra) are distributed throughout the NT of hAtg3 and the C-terminal part of the protein, as shown in Fig. 5b. The large shifts for hAtg3 NT residues are expected since they undergo transition from a random coil to an α-helix structure as demonstrated by secondary ^13^C_α_ chemical shifts for residues 3–19 in aqueous solution and in bicelles (Supplementary Fig. 14). On the other hand, large CSPs for resonances from the C-terminal part of the protein (such as Y220, S237, E248, and H252 in Fig. 5a) suggest that structural rearrangements beyond hAtg3’s NT occur upon interaction with the bilayer. In Fig. 5c, the perturbed (cyan) and unperturbed (orange) residues are highlighted on an hAtg3 structural model; unassigned residues are colored white. Interestingly, perturbed and unassigned residues congregate on one side around the active site (C264) while the unperturbed residues cluster on the opposite side.

**Fig. 6:**
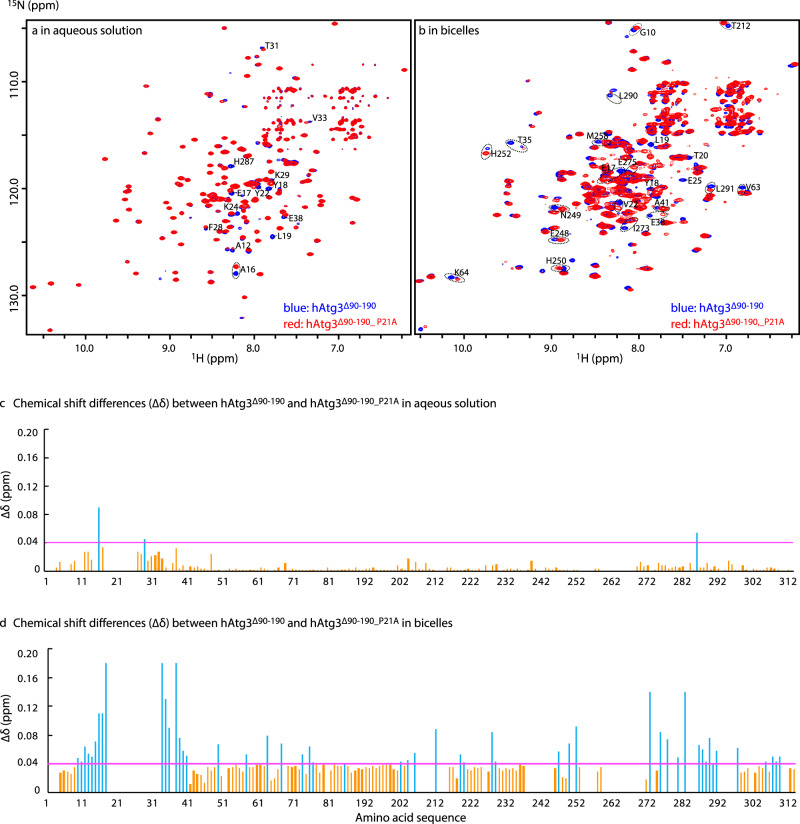
Binding of hAtg3^∆90-190_P21A^ to bicelles induces structural changes different from the wild-type protein. **a**, **b** Overlay of TROSY spectra of ^2^H,^15^N,^13^C-labeled hAtg3^∆90-190^ (blue) and hAtg3^∆90-190_P21A^ (red) in aqueous solution (pH 7.5) and in bicelles (DMPC:DMPG:DHPC = 4:1:20, molar ratio, pH 7.5), respectively. Both spectra were acquired on a Bruker 600 MHz spectrometer at 25 °C. **c** and **d** Plots of chemical shift differences (∆δ) between hAtg3^∆90-190^ and hAtg3^∆90-190_P21A^ against residue numbers in aqueous solution and in bicelles, respectively.

To understand why the hAtg3^P21A^ mutant fails to catalyze the transfer of LC3B from LC3B-hAtg3 to PE in the membrane despite having wild-type-like membrane binding and thioester intermediate formation, we examined its behavior in the membrane-bound state. Figure 6a, b displays overlays of TROSY spectra of hAtg3^∆90-190^ and hAtg3^∆90-190_P21A^ in aqueous solution and in bicelles. In the absence of bicelles, the two spectra overlap well and all shifted resonances occur near the mutation site except H287, which is located at the beginning of the last α-helix (Fig. 6a, c). This indicates only local structural changes. Conversely, in bicelles there are extensive perturbations in both the NT and C-terminal portions of hAtg3^∆90-190_P21A^ compared to the wild type (Fig. 6b, d), illustrating that while hAtg3^∆90-190_P21A^ also undergoes a conformational shift upon membrane binding, the structure of membrane-bound hAtg3^∆90-190_P21A^ differs from the structure of membrane-bound hAtg3^∆90-190^. This suggests that the loss of conjugase activity of hAtg3^P21A^ may be due to its altered membrane-bound structure. Additionally, as expected, hAtg3^∆90-190_V8D_V15K^, a mutant that is unable to bind the membrane and has no LC3B–PE conjugation activity (Fig. 3), shows minimal conformational shifts in bicelles (Supplementary Fig. 15). Thus, together, our NMR data show that the binding of the hAtg3 N-terminal AH to the membrane results in structural rearrangements in the C-terminal part of the protein around its active site and furthermore, the NT^Cons^ region of hAtg3 is a key mediator of this structural transition.

## Discussion

This study reveals an expanded role for the hAtg3 NT in the regulation of LC3B–PE conjugation. In isolation, human and yeast NTs have similar membrane-geometry sensitivity, but they are not functionally exchangeable; swapping their NTs (hAtg3^yNT^) results in a nearly complete loss of its catalytic activity. This is due to the NT^Cons^ region, rather than the membrane-interacting region where there is sequence divergence between homologs. Indeed, we found that mutations of the conserved Pro21, or other residues in its vicinity, had deleterious effects on LC3B–PE conjugation despite the fact that proteins harboring these mutations bind to the membrane and form LC3B-hAtg3 intermediates normally. This indicates that while the N-terminal AH is necessary for targeting hAtg3 to the membrane, it is not sufficient for LC3B–PE conjugation. For LC3B–PE conjugation to occur, the hAtg3 NT^Cons^ region must convey its membrane geometry-dependent interactions to its active site, as evidenced by NMR CSP data. The essential function of the hAtg3 NT^Cons^ region is also demonstrated in vivo by the hAtg3^P21A^ and hAtg3^L23T^ mutants with a caveat that decreased Atg12–Atg5 conjugation has been reported previously in Atg3 knockout cells^29^.

Direct structural information for the LC3-Atg3 intermediate, its membrane-bound activated form, and the mechanism of activation remains elusive. The current model for Atg3 activation is based on an analysis of the yAtg3 structure crystallized at pH 5.8 (assumed to be an inactive state)^11^, the AtAtg3 (*A. thaliana* Atg3) structure crystallized at pH 8.0 (assumed to be an active state)^9^, and Cys cross-linking data by the Ohsumi group^13^. In this model, interactions between yAtg3 and Atg5-Atg12/Atg16 induce a structural rearrangement of the active site of yAtg3, which presumably involves the catalytic Cys234 orienting toward a Thr213, similar to the configuration seen in the active site of an E2 ligase^30–32^. Furthermore, a recent study showed that yAtg3 is autoinhibited, and identified an allosteric switch (residues 129–142) that may mediate a conformational rearrangement in its active site upon interacting with the Atg5–Atg12/Atg16 complex^26^. Our studies indicate that an additional structural arrangement occurs in and around the active site of hAtg3 due to its N-terminal AH binding to the membrane. This structural change, which is presumably distinct from that induced by Atg5–Atg12/Atg16 binding, is functionally critical for transferring LC3B from LC3B-hAtg3 to PE in the membrane. Mutants such as hAtg3^P21A^, which show altered structural rearrangements after membrane binding catalyze little LC3B–PE conjugation. We speculate that upon the binding of hAtg3’s AH to the membrane, the NT^Cons^ region triggers a conformational change that brings the thioester LC3B-hAtg3 intermediate to the membrane surface in close proximity to substrate PE lipids and orients it optimally for the transfer of LC3B to PE. Although this study has been focused on LC3B, the membrane-induced structural arrangements may also be required for the lipidation of other members of the Atg8 family since the same chemistry is used to catalyze their conjugations to PE lipids. On the other hand, recent studies have revealed that each of the LC3/GABARAP superfamily members may have a distinct role in the process of autophagy^33,34^. It is conceivable that the N-terminal region of Atg3 might influence the lipidation reaction among the family members differently, and this remains to be examined.

Membrane interactions that lead to changes in protein structure and activity have been reported in other systems, such as Arf1 and ArfGAP1. In Arf1, the binding of its N-terminal AH to the positively curved membrane results in a conformational change in its active site for GTP loading^35^. For ArfGAP1, the activation of the protein depends on the insertion of its ALPS motif into the highly curved membrane^36^ although the structural and molecular mechanism of this activation is not yet known. To understand how the interaction of hAtg3 with the membrane contributes to its activity, high-resolution structural information on its membrane-bound active states is necessary. We are working toward this goal despite the challenges inherent in obtaining this data. Atg3 has been referred to as an intrinsically disordered protein^37^ and its catalytic site in the apo-state is in a constant state of conformational exchange as described above. In the TROSY spectrum of hAtg3 bound to bilayer-like bicelles, many resonances around the active site suffer from exchange broadening which complicates their backbone resonance assignment and detailed NMR structural analysis.

In summary, we have demonstrated that in hAtg3, the membrane geometry-sensitive AH and conserved region of the NT work together to promote LC3B–PE conjugation only on the target membrane. While future studies are required to fully understand the mechanism of action, our results support an emerging concept that membrane geometry provides an essential cue for spatial regulation of autophagosome biogenesis during autophagy^38–41^.

## Methods

### Atg3 peptides

Atg3 human (MQNVINTVKGKALEVAEYLTPVLKESK) and yeast (MIRSTLSSWREYLTPITHKST) NTs and hAtg3 NT mutants (A12W, A12W_P21A, A12W_L23T, A12W_V8D_V15K) were synthesized by GenScript (Piscataway, New Jersey) at >95% purity as determined by high-performance liquid chromatography (HPLC) and mass spectroscopy (MS). Peptides were lyophilized in order to further remove trace organic solvents. Peptide stock solutions were prepared at a concentration of 1 mg/mL by weighing lyophilized peptide and the concentrations were determined by a NanoDrop spectrophotometer (Thermo Scientific, Waltham, MA). The yAtg3 NT peptide was prepared by dissolving the peptide in H_2_O. The hAtg3 NT peptides were prepared by dissolving the peptide in 0.1% HCl in water. Peptides were stored at −20 °C before use.

### Preparation of liposomes, bicelles, and nanodiscs

All lipids were purchased from Avanti Polar Lipids, Inc., including POPC (1-palmitoyl-2-oleoyl- sn-glycero-3-phosphocholine), DOPG (1,2-dioleoyl-sn-glycero-3-phospho1’-rac-glycerol), DOPE (1,2-dioleoyl-sn-glycero-3-phosphoethanol-amine), DMPC (1,2-dimyristoyl-sn-glycero-3-phosphocholine), DMPG (1,2-dimyristoyl-sn-glycero-3-phospho-(1’-rac-glycerol) (sodium salt)), DHPC (1,2-dihexanoyl-sn-glycero-3-phosphocholine), LPC (1-myristoyl-2-hydroxy-sn-glycero-3-phosphocholine), LPG (1-myristoyl-2-hydroxy-sn-glycero-3-phospho-(1’-rac-glycerol) (sodium salt)), and LPE (1-myristoyl-2-hydroxy-sn-glycero-3-phosphoethanolamine).

For liposome preparation, lipids in chloroform were added to a glass tube and dried to a thin film by spinning with heat for an hour using a condenser rotor SpeedVac, followed by lyophilization overnight once the volatile organics were removed. Lipids were rehydrated with H_2_O for 1 h at 42 °C and vortexed every 15 min. Rehydrated lipids were transferred to a 1.5 mL plastic tube and put through 5 cycles of freeze-thaw using a dry ice bath and a room temperature water bath. The mixture was then transferred to a clean glass tube for bath sonication (BRANSON 3510R-MT Bransonic Ultrasonic Cleaner). The lipids were sonicated for 15-min intervals until clear, then stored at room temp. If liposomes were not consumed within 2 days, they were sonicated for an additional 5–10 min before use. Extruded liposomes were prepared as described previously^42^. Briefly, lipids were prepared as above, but instead of sonication, the rehydrated lipids were passed through a 0.05 μm filter 11 times (SPARTAN HPLC Syringe Filter) in a liposome extruder (Avanti Polar Lipids) after the dry ice bath. The extruded liposomes were collected and stored at room temperature.

Bicelles and nanodiscs (cNW9, circulated nanodisc width of 9 nm) were prepared as described previously^43–45^. Briefly, for the preparation of bicelles, DMPC, DMPG, and LPE were mixed together with double distilled H_2_O (ddH_2_O), frozen on ethanol/dry ice, and thawed at 42 °C for 3 freeze-thaw cycles. DHPC in ddH_2_O was then added into the mixture and quickly vortexed to form a clear solution. The solution was frozen on ethanol/dry ice and slowly thawed at room temperature for use. For the preparation of nanodiscs, purified NW9 was circularized to form cNW9 (circularized NW9) using freshly made sortase in buffer (300 mM Tris-HCl, pH 7.5, 150 mM NaCl, 10 mM CaCl_2_). cNW9 was purified through Ni column and then S200 16/60 column. The purified cNW9 was exchanged into a buffer of 20 mM Tris, 100 mM NaCl, pH 8.0, and 20 mM sodium cholate, and incubated with lipids (POPC/POPE/POPG in a molar ratio of 2/2/1, cNW9/lipid in a molar ratio of 1/60) on ice for 1 h. Sodium cholate was then removed by incubation with Bio-beads SM-2 (Bio-Rad) for 1 h on ice followed by incubation overnight at 4 °C. Bio-beads were removed through 0.22 μm nitrocellulose filter. The nanodisc preparations were further purified by size-exclusion chromatography and assessed using SDS–PAGE.

### Circular dichroism (CD) spectroscopy

CD spectroscopy was performed on Jasco 710 and 1500 J-spectropolarimeters. Samples contained 0.1 mg/mL of hAtg3 or yAtg3 NT peptides in a buffer of 20 mM Tris HCl, pH 7.5, and 50 mM NaCl with and without liposomes in a 0.1 mm path length cuvette. CD spectra were collected for 200–250 nm at 25 °C. Each spectrum was collected by averaging three scans at a rate of 50 nm/min and 1 nm or 0.2 nm step size. After recording, the spectra were subjected to background subtractions and baseline corrections.

### Protein expression and purification

Human Atg3 (hAtg3) and LC3B (with Gly120 as the last amino acid) were subcloned into the pET28a expression vector at the BamHI/XhoI site with His-tag and T7-tag and a thrombin cleavage site at the N-termini, and plasmids were transformed into chemically competent Rosetta™(DE3) pLysS cells for expression. All hAtg3 mutants were generated using the Q5 Site-Directed Mutagenesis Kit (New England Biolabs) or the QuickChange MultiSite-Directed Mutagenesis Kit (New England Biolabs) with corresponding primers listed in Supplementary Table 1 and verified by sequencing. Typically, a single colony was selected to grow in small volume of LB medium overnight at 37 °C as a starter culture and then inoculated into a large volume of LB medium (for unlabeled proteins) or M9 medium supplemented with D-glucose (or 3 g/L D-glucose-^13^C_6_ and D_2_O) and ^15^NH_4_Cl (1 g/L) for ^15^N (or ^15^N/^13^C/^2^H) labeled samples at 37 °C until OD_600_ reached 0.8. The temperature was then lowered to 25 °C and cells were induced with 0.5 mM IPTG for ~16 h. Cells were harvested by centrifugation and the pellets were stored at −80 °C until use. Mouse Atg7 (mAtg7) was subcloned into the pFast-BacI baculovirus vector at the StuI/XbaI sites with a His-tag at the N terminus and expressed in High Five insect cells^46^.

Cell pellets (for hAtg3, LC3B, or mAtg7) were homogenized with a lysis buffer of 20 mM phosphate, pH 7.5, 300 mM NaCl, 2 mM beta mercaptoethanol (BME), and 1 mM MgCl_2_ supplemented with benzonase nuclease and complete protease inhibitor cocktail (Roche), and were lysed using sonication on ice with 2 s on/7 s off intervals for 18 min total duration. Cell debris was removed by centrifugation (Sorvall RC5B Plus Refrigerated Centrifuge) at 26,900*g* at 10 °C for 30 min. Supernatants were collected, and either incubated with HisPur Ni-NTA resin and applied to a gravity column or loaded onto a Ni-NTA column (HisTrap HP). The column was washed with PBS buffers (20 mM phosphate, pH 7.5, 300 mM NaCl, 2 mM BME) without and with 30 mM imidazole, and then eluted with PBS buffer containing 500 mM imidazole. For biochemical assays, the elute of hAtg3 was concentrated and exchanged to a low-salt buffer of 20 mM Tris, pH 8.0, and 2 mM BME, and then loaded onto a 1 mL Capto Q ion exchange column (GE Healthcare Life Sciences). The column was washed with a buffer of 50 mM HEPES, pH 8.0, 2 mM BME, and targeted protein was eluted with a rising gradient of 50 mM HEPES, 1 M NaCl, and 2 mM BME until no more protein was indicated by the UV measurements. Relevant fractions with purities no less than 90% were pooled after analysis on an SDS-PAGE gel. For NMR experiments, the elute of hAtg3 from Ni-column was concentrated and exchanged to a buffer (10 mL) containing 50 mM HEPES, pH 7.5, 150 mM NaCl, and 2 mM BME, and followed by the addition of 0.1% (v/v) TWEEN20 and 50 units thrombin to remove T7-tag and His-tag for an overnight agitation at 4 °C. The solution was then subjected to a Q-column and further purified by size-exclusion chromatography using an S75 column (HiLoad 16/60 Superdex 75) and a buffer of 50 mM HEPES, 1 M NaCl, and 1 mM DTT. For LC3B and Atg7, elutes from the Ni-column were concentrated and subjected to S75 column purification. Purified hAtg3 or mutant proteins, LC3B and mAtg7 were exchanged into a buffer of 50 mM HEPES, pH 7.5, 150 mM NaCl, and 2 mM TCEP (tris(2-carboxyethyl)phosphine). Protein concentration was assayed using a Nanodrop (Thermo Scientific, Waltham, MA).

### In vitro conjugation assay

The in vitro conjugation was conducted similarly to previous descriptions^15^. Typically, 2.5–5.0 μM hAtg3 or mutant (with a His tag and T7 tag at the N-termini^47^) was combined with 5 μM LC3B, 0.5–1 μM mAtg7, and 1 mM MgCl_2_ in a reaction buffer (50 mM HEPES, 150 mM NaCl, 2 mM TCEP, and pH 7.5) of 37.5 μL. First, 3.7 μL were removed for a control, then 1 mM ATP was added and the reaction was allowed to proceed for 30 min at 37 °C for intermediate formation. Then, 3.75 μL were removed to check for intermediates, and an additional 3.75 μL were removed as a non-liposome containing control. Then, 1 mM liposomes were added to the reaction system (final volume of 35 μL). The reaction was allowed to proceed at 37 °C with gentle agitation, with 4.5 μL samples removed at 0, 15, 30, 60, 90, 120, and 180 min. Samples were mixed with 4 μL 4X protein loading buffer (10% w/v SDS, 10% BME, 40% Glycerol, 250 mM Tris-HCL, pH 6.8, and 0.4% bromophenol blue dye), frozen immediately to stop the reaction, and stored at −80 °C until analyzing with 18% polyacrylamide gel electrophoresis. For gel electrophoresis, samples were heated at 95 °C for 3 mins. Gels were stained with SimplyBlue SafeStain (Invitrogen) until bands became discernable. Gels were destained in deionized water overnight, imaged on a BioRad Chemidock MP imager, and analyzed using ImageJ.

### Liposome co-sedimentation assay

First, 2 μM hAtg3 (or its mutants) was mixed with 800 μM sonicated liposomes (POPC/DOPG/DOPE with a molar ratio of 3:2:5) or without liposomes as a control in a buffer A of 50 mM HEPES, 150 mM NaCl, and 2 mM TCEP. Then, the mixture of a total volume 300 μL was incubated at 37 °C for 1 h, and 12 μL were removed and mixed with 4 μL 4X protein loading buffer (total, T). The remaining mixture was subjected to ultracentrifugation at 160,000*g* for 2 h at 4 °C (Optima MAX Ultracentrifuge). After removing the supernatant, the pellets were gently washed once with 288 μL buffer A and resuspended in 288 μL buffer A. Then, 12 μL of the supernatant (S) and the pellet resuspension (P) were mixed with 4 μL 4X protein loading buffer, respectively, for gel electrophoresis (NuPAGE 10% Bis-Tris). Samples were heated at 95 °C for 3 mins for gel electrophoresis.

### Mammalian cell culture

HEK293T cells (ATCC; CRL-3216) and Atg3^−/−^ MEFs provided by Dr. Shengkan (Victor) Jin (Rutgers University - Robert Wood Johnson Medical School, NJ) were cultured in Dulbecco’s Modification of Eagle’s Medium (DMEM) supplemented with 10% fetal bovine serum and 1x Antibiotic Antimycotic Solution (AA) (Corning, 30-004-CI).

### Plasmid construction

hAtg3, hAtg3^P21A^, and hAtg3^L23T^ cDNAs were amplified by PCR (forward: 5′-GAGCTGTACAAGTCTAGAGTGATGCAGAATGTGATTAATA-3′; reverse 5′-CGCAGATCCTTGCGGCCGCGTTACATTGTGAAGTGTCTTG-3′). Purified PCR products were subcloned into pCDH1-CMV-mCherry-MCS-EF1-puro viral backbone using NheI and BamHI.

### Lentiviral packaging, transduction, and cell sorting

HEK293T cells were transfected with Invitrogen ViraPower lentiviral packaging plasmids (pLP1, pLP2, and pLP/VSVG) and viral vector using Lipofectamine 3000 per the manufacturer’s protocol. Culture medium was replaced 6 h after transfection; viral supernatant was collected 24 and 52 h post-transfection and subjected to 0.45 μm filtration. Atg3^−/−^ MEFs were incubated with filtered viral supernatant for 24 h and subjected to puromycin selection 48 h post-transduction for 3 days. The transduction was repeated three times, followed by flow cytometry sorting based on mCherry intensity. The cells were divided into three intensity levels of low, medium, and high. MEF cells expressing medium mCherry intensity were used for autophagic flux assay.

### Autophagic flux assay

Cells were incubated in complete media or starvation media (amino-acid and serum free) in the presence or absence of 100 nM bafilomycin A1 (BafA1) for 3 h and subjected to immunoblotting using the mCherry antibody (Abcam, ab125096) with a ratio of 1:1000, the β-actin antibody (Sigma, A5441-100UL) with a ratio of 1:10,000, and the LC3 antibody (Novus Biologicals, NB100-2220) with a ratio of 1:5,000. To quantify the LC3-II level, the experiments were repeated six times. The signal intensity of LC3-II was measured using Image Studio version 5 software (LI-COR Biotechnology), then normalized to the intensity of β-actin of the same sample. To calculate the relative LC3-II level, the LC3-II value of Atg3^−/−^ MEFs expressing mCherry-hAtg3 in starvation medium with BafA1 was set to be 1. The autophagic flux was calculated by subtracting the LC3-II value in the absence of BafA1 from that in the presence of BafA1 in complete (basal) or starvation (induced) medium. All the values were then normalized with the value of Atg3^−/−^ MEFs expressing mCherry-hAtg3 being set as 1. The data were analyzed by one-way ANOVA test followed by Dunnett’s multiple comparisons test using GraphPad Prism7.0.

### NMR spectroscopy

Typical NMR samples contained 0.3–0.5 mM labeled protein in a buffer of 25–50 mM HEPES, pH 6.5 (or 7.5), 150 mM NaCl, 2 mM TCEP, and 0.02% NaN3. For samples with 12% bicelles, the pH was at 7.5 since the protein precipitates after a few days at pH 6.5. All NMR data were acquired at 25 °C on Bruker 600 or 850 MHz spectrometers equipped with cryoprobes. The data were processed using NMRPipe and analyzed using NMRView. Backbone and ^13^C_β_ resonance assignments were carried out using TROSY-based triple resonance HNCO, HN(CA)CO, HNCA, HN(CO)CA, HNCACB, and HN(CO)CACB experiments. Typical data acquisition parameters are listed in Supplementary Table 2. The CSPs of ^1^H and ^15^N backbone resonances were calculated as ${{\Delta }}\delta = \sqrt {0.5 \ast \{ {( {\frac{{\delta _{\mathrm{N}}}}{5}} )^2 + \delta _{\mathrm{H}}^2} \}}$, $\delta _{\mathrm{H}}$ and $\delta _{\mathrm{N}}$ represent the changes in ^1^H and ^15^N chemical shifts^48^.

### Reporting summary

Further information on research design is available in the Nature Research Reporting Summary linked to this article.

## Supplementary information

Supplementary Information

Peer Review File

Reporting Summary.

## Data Availability

NMR resonance assignments have been deposited with the BMRB (www.bmrb.io) with accession numbers 50479 (hAtg3^∆90-190^ in aqueous solution), 50480 (hAtg3^∆90-190^ in bicelles), 50481 (hAtg3^∆90-190_P21A^ in bicelles), and 50470 (hAtg3^∆1-25^ in aqueous solution). PDBs of 3VX8 [10.2210/pdb3VX8/pdb], 6OJJ [10.2210/pdb6OJJ/pdb], and 2DYT [10.2210/pdb2DYT/pdb] are available at www.rcsb.org. All other data and materials that support the findings of this study are available from the corresponding authors upon request. Source data are provided with this paper.

## Source data

Source Data
