# Supplementary material for: An N-terminal conserved region in human Atg3 couples membrane curvature sensitivity to conjugase activity during autophagy
Source: Nat Commun. 2021 Jan 14;12:374. doi: 10.1038/s41467-020-20607-0 (PMC7809043; doi:10.1038/s41467-020-20607-0)
Supplement: Supplementary file 1 — Supplementary Information [file 41467_2020_20607_MOESM1_ESM.pdf]

Supplementary Information

An N-terminal conserved region in human Atg3 couples membrane curvature sensitivity to conjugase activity during autophagy

Yansheng Ye[1*], Erin R. Tyndall[1*], Van Bui[2*], Zhenyuan Tang[2], Yan Shen[3], Xuejun Jiang[4], John M. Flanagan[1], Hong-Gang Wang[2,§], and Fang Tian[1,§]

[1]Departments of Biochemistry and Molecular Biology, Penn State College of Medicine, Hershey, PA, USA

[2]Departments of Pediatrics, Penn State College of Medicine, Hershey, PA, USA

[3]Laboratory of Chemical Physics, National Institute of Diabetes and Digestive and Kidney Diseases, US National Institute of Health, Bethesda, Maryland, USA

[4]Cell Biology Program, Memorial Sloan Kettering Cancer Center, New York, NY, USA

* These authors contributed equally to this work,

[§] To whom correspondence should be addressed: Hong-Gang Wang (huw11@psu.edu) or Fang Tian (ftian@psu.edu).

Supplementary Table 1: Primers used in this study

**yNT**	F	5'	GGCTGAGTACCTGACCCCGGTCCTCAAGGAATCAAAGTTTTTAACCACAGGTCAAATAACTCC
	R	5'	ACTTCCAGTGCCTTTCCCTTCACAGTATTAATCACATTCTGCATATGGTCGACCCGGGA
**yNTvar**	F	5'	GAGCAGCTGGCGTGAGTACCTGACCCCGGTC
	R	5'	AGGGTGCTACGAATCATGGATCCGCGACCCAT
**yNTcons**	F	5'	AAAAGCACCTTTAAGGAAACAGGTGTAATTACCC
	R	5'	GTGGGTAATCGGGGTCAGGTACTCAGC
**V8D**	F	5'	CATGCAGAATGTGATTAATACTGATAAGGGAAAGGCACTGGAAG
	R	5'	
**V15K**	F	5'	GGAAAGGCACTGGAAAAGGCTGAGTACCTGACCC
	R	5'	
**P21A**	F	5'	GTACCTGACCGCTGTCCTCAAGGAATC
	R	5'	TCAGCCACTTCCAGTGCC
**L23T**	F	5'	GACCCCGGTCACCAAGGAATCAAAG
	R	5'	AGGTACTCAGCCACTTCC
**E25K**	F	5'	GGTCCTCAAGAAGTCAAAGTTTAAGGAAACAG
	R	5'	GGGGTCAGGTACTCAGCC
**+7Gly**	F	5'	GGAGGTGGAGGCTTTAAGGAAACAGGTG
	R	5'	ACCTCCACCCTTTGATTCCTTGAGGAC
**90-190 deletion**	F	5'	GAAGATGCTATTTTGCAAACC
	R	5'	TTCCATCTGTTTGCACCG
**His-tag thrombin site**	F	5'	TGCTAGCCATATGCCTGTCGTGCGGCACCAGGCC
	R	5'	GGCCTGGTGCCGCACGACAGGCATATGGCTAGCA
**T7-tag thrombin site**	F	5'	TCTGCATGGATCCGCGAGGCACTAGCTGTCCACCAGTCATG
	R	5'	CATGACTGGTGGACAGCTAGTGCCTCGCGGATCCATGCAGA
**NT thrombin site**	F	5'	CTGTTTCCTTAAACTTTGATCCCCTGGGGACCAGGGTCAGGTACTCAGCCAC
	R	5'	GTGGCTGAGTACCTGACCCTGGTCCCCAGGGGATCAAAGTTTAAGGAAACAG

Supplementary Table 2: Typical data acquisition parameters for TROSY-based triple resonance experiments (Salzmann, M. et al. (1999), J. Am. Chem. Soc. 121: 844-848; Schulte-Herbrueggen, T. (2000), J. Magn. Reson.144: 123-128; Eletsky, A. et al. (2001), J. Biomol. NMR 20: 177-180)

Experiments	t3 dimension (^{1}H)	t2 dimension (^{15}N)	t1 dimension (^{13}C)	Number of scans (1.5 s recycle delay)	
	Spectral width / Complex points	Spectral width / Complex points	Spectral width / Complex points	Aqueous solution	Bicelle solution
HNCO	9615.4 Hz	1824.3 Hz	2263.7 Hz	16	16
	1024	28	30		
HNCACO	9615.4 Hz	1824.3 Hz	2263.7 Hz	16	32
	1024	28	30		
HNCA	9615.4 Hz	1824.3 Hz	4075 Hz	16	48
	1024	28	24		
HNCOCA	9615.4 Hz	1824.3 Hz	4075 Hz	16	48
	1024	28	24		
HNCACB	9615.4 Hz	1824.3 Hz	9355.3 Hz	32	48
	1024	28	40		
HNCOCACB	9615.4 Hz	1824.3 Hz	9355.3 Hz	32	48
	1024	28	40		

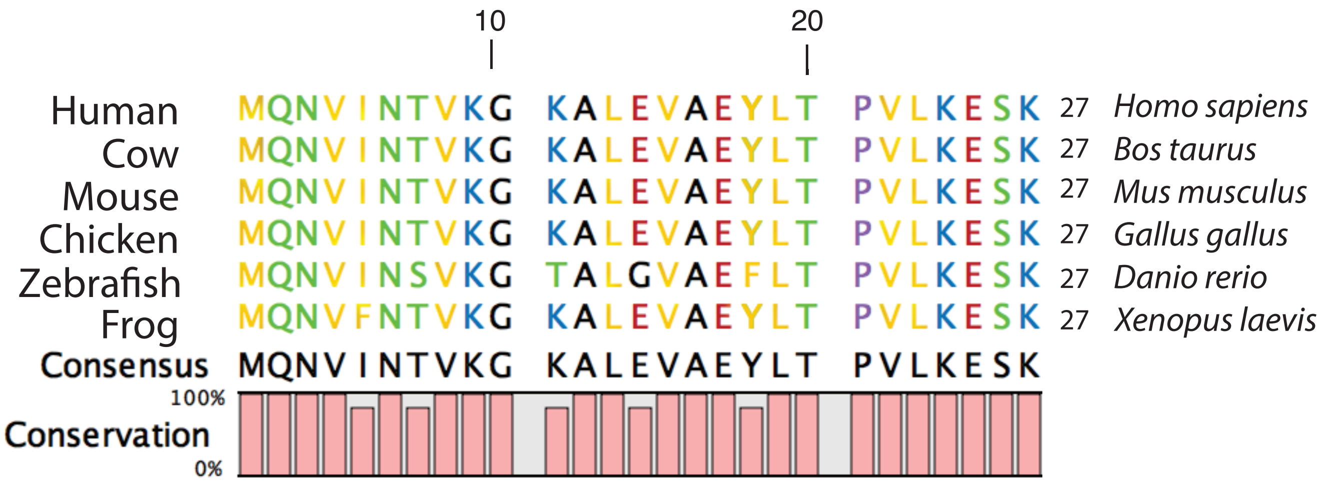

Supplementary Figure 1: Sequence comparison of Atg3 animal homologs. Non-polar and aromatic residues are in yellow, polar residues in green, positively charged in blue, negatively charged residues in red, Ala and Gly residues in black, and Pro residue in purple.

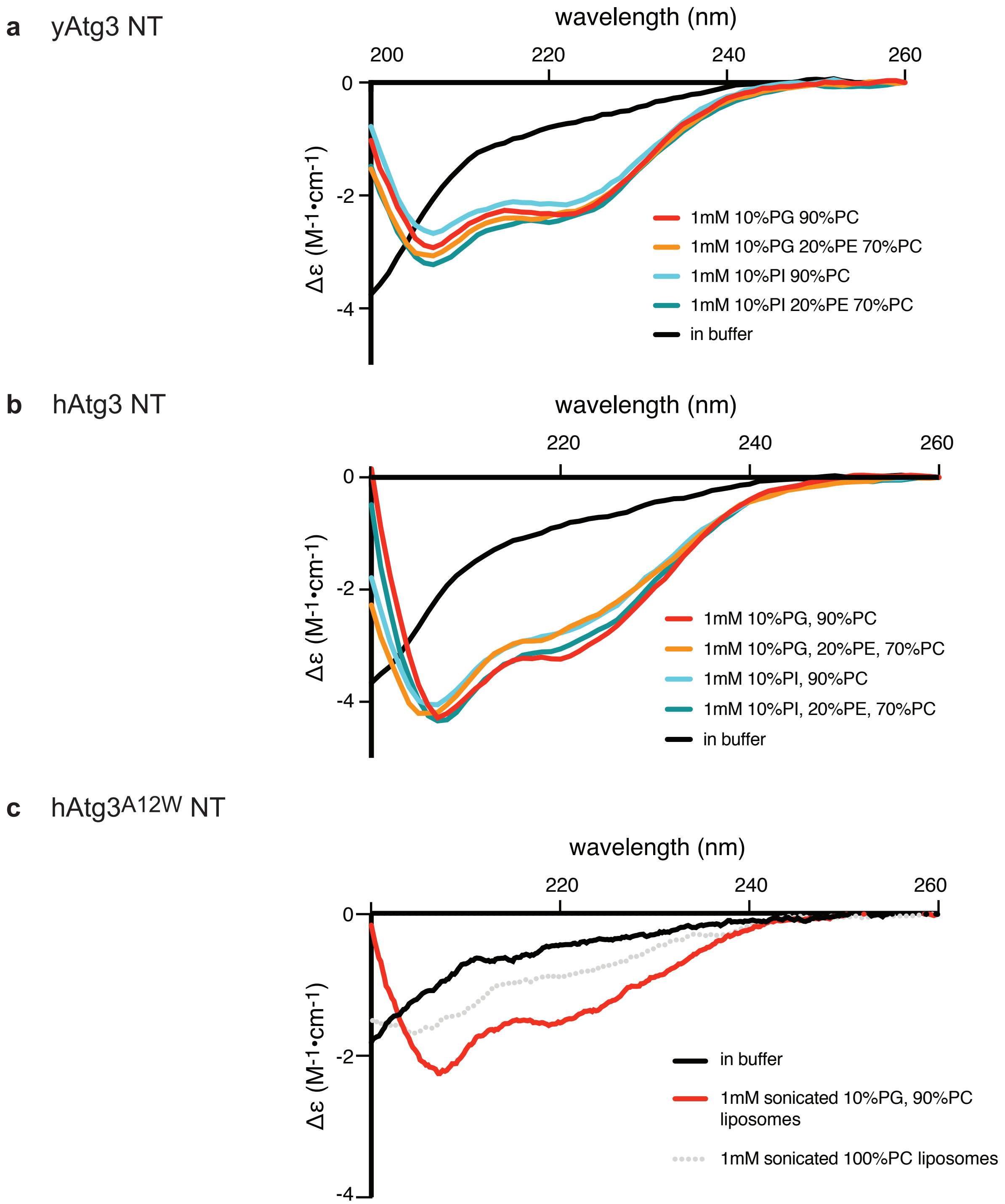

Supplementary Figure 2: Structural rearrangements to an α-helical conformation are not sensitive to exact lipid composition and require the presence of negatively charged lipids (PGs or PIs), but not PEs. CD spectra of yAtg3 NT (a) and hAtg3 NT (b) in the absence and presence of sonicated liposomes with various compositions consisting of the described ratios of POPC, DOPG and DOPE. (c) CD spectra of hAtg3^{A12W} NT in the presence of buffer, sonicated 10%DOPG, 90% POPC liposomes, and sonicated 100% POPC liposomes. To facilitate the purification and quantification of hAtg3 NT, an Ala to Trp mutation at residue 12 is introduced. This mutation does not affect the folding of hAtg3 NT.

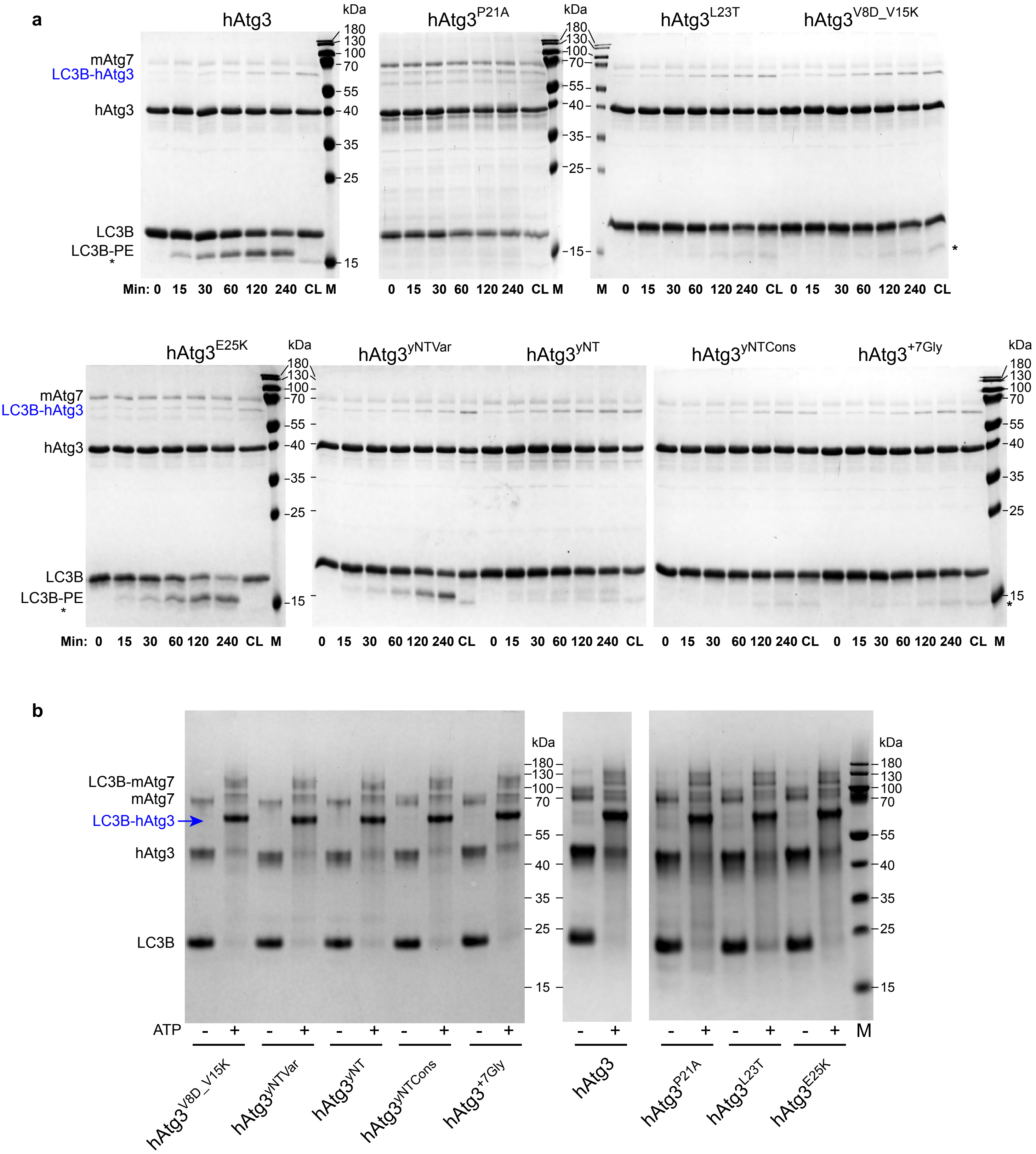

Supplementary Figure 3: (a) Representative full SDS-PAGE images of reaction mixtures composed of hAtg3 and its P21A, E25K, L23T, V8D_V15K, yNTVar, yNT, yNTCons and seven Gly insertion (+7Gly) mutants (5 μM), mAtg7 (mouse Atg7, 0.5 μM), LC3B (5 μM), ATP (1 mM) and PE-containing liposomes (1 mM) (details in Methods). CL is the control without liposomes, and M is for the marker. The asterisk indicates a small amount degradation of LC3B in the presence of ATP, which runs slightly faster than LC3B-PE. (b) NuPAGE (10% Bis-Tris, Invitrogen) images of hAtg3 and its mutants (5 μM) incubated with mAtg7 (0.5 μM), LC3B (5 μM) with (+) and without (-) ATP (1 mM) for 30 min at 37 ^{o}C. The formation of intermediate is indicated by blue arrow. All experiments were repeated three times (n=3).

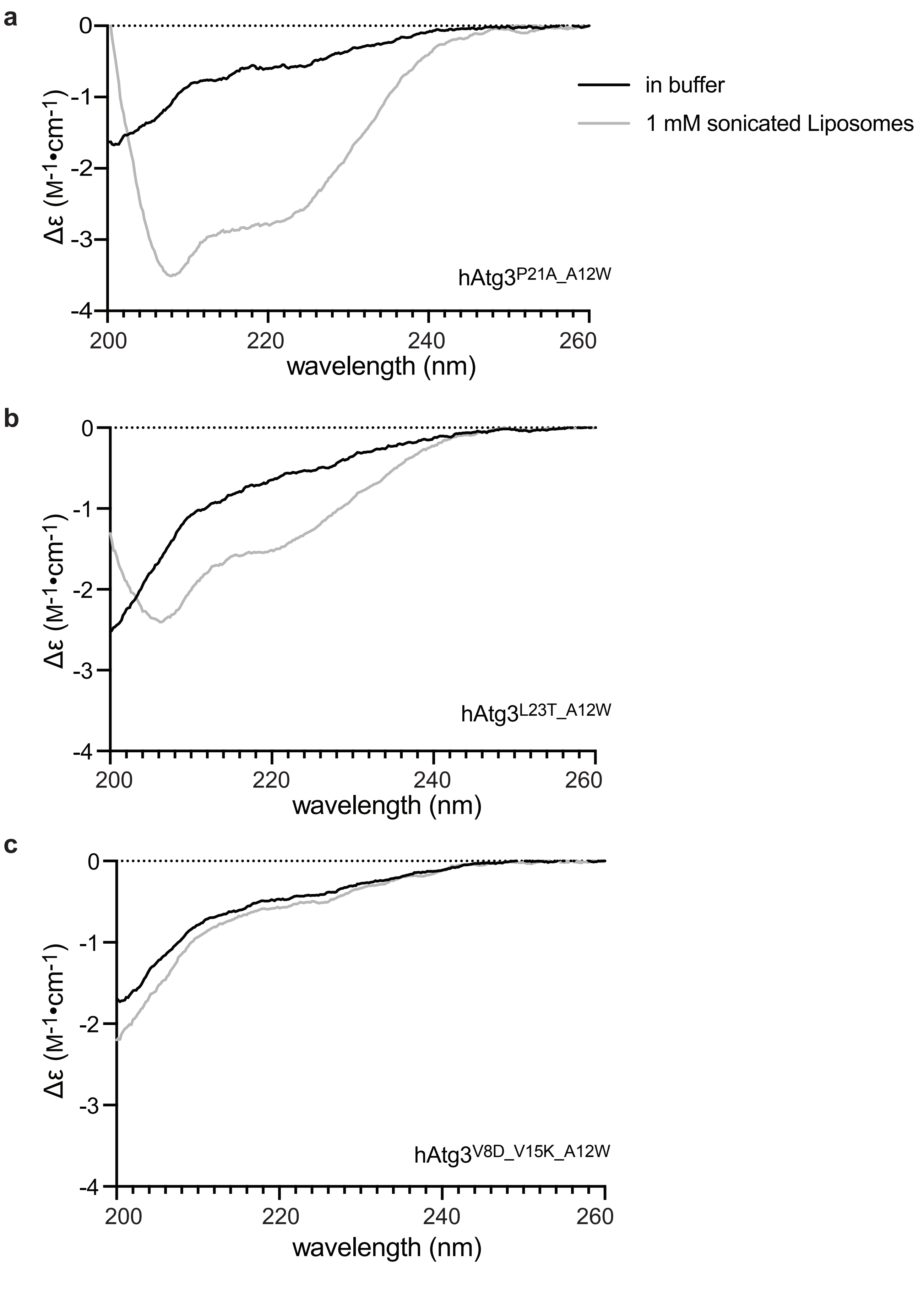

Supplementary Figure 4: CD spectra of $hAtg3^{P21A\_A12W}$, $hAtg3^{L23T\_A12W}$, and $hAtg3^{V8D\_V15K\_A12W}$ NT peptides in the absence and presence of 1 mM 10% DOPG, 90% POPC sonicated liposomes.

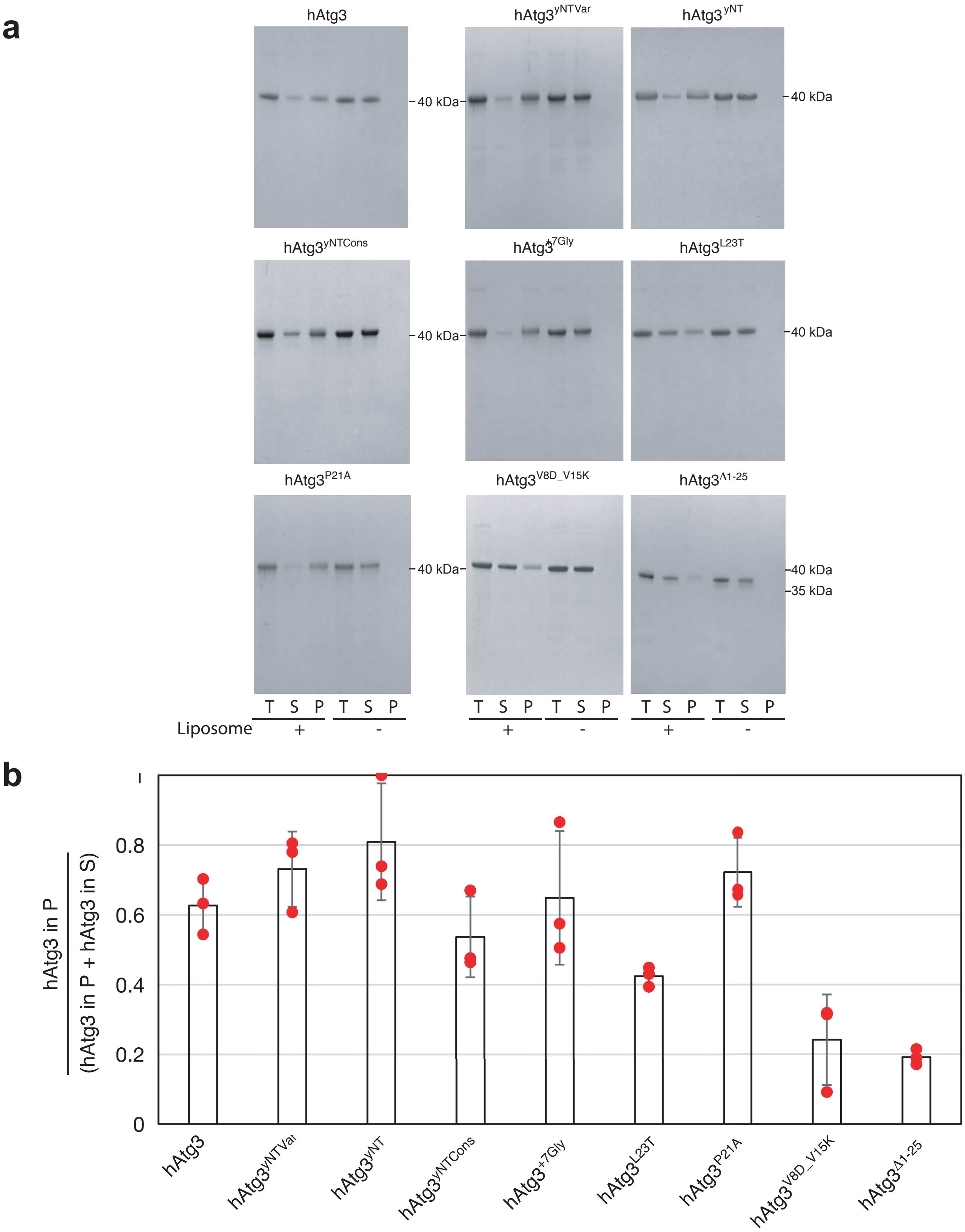

Supplementary Figure 5: Interaction of hAtg3 and its mutants with liposomes in co-sedimentation assay. (a) Binding of hAtg3 and its mutants to liposomes. hAtg3 and its mutants (2 μM) were incubated with (+) and without (-) liposomes (800 μM) at 37 °C for 1 hr. The liposome-associated hAtg3 and its mutants were pelleted down by ultracentrifugation and analyzed by NuPAGE (10% Bis-Tris, Invitrogen). T: total; S: supernatant; P: pellet. (b) Fraction of the amount of hAtg3 and its mutants in the pellet. Error bars indicate SD (standard deviation) from three independent experiments (n=3 for each construct).

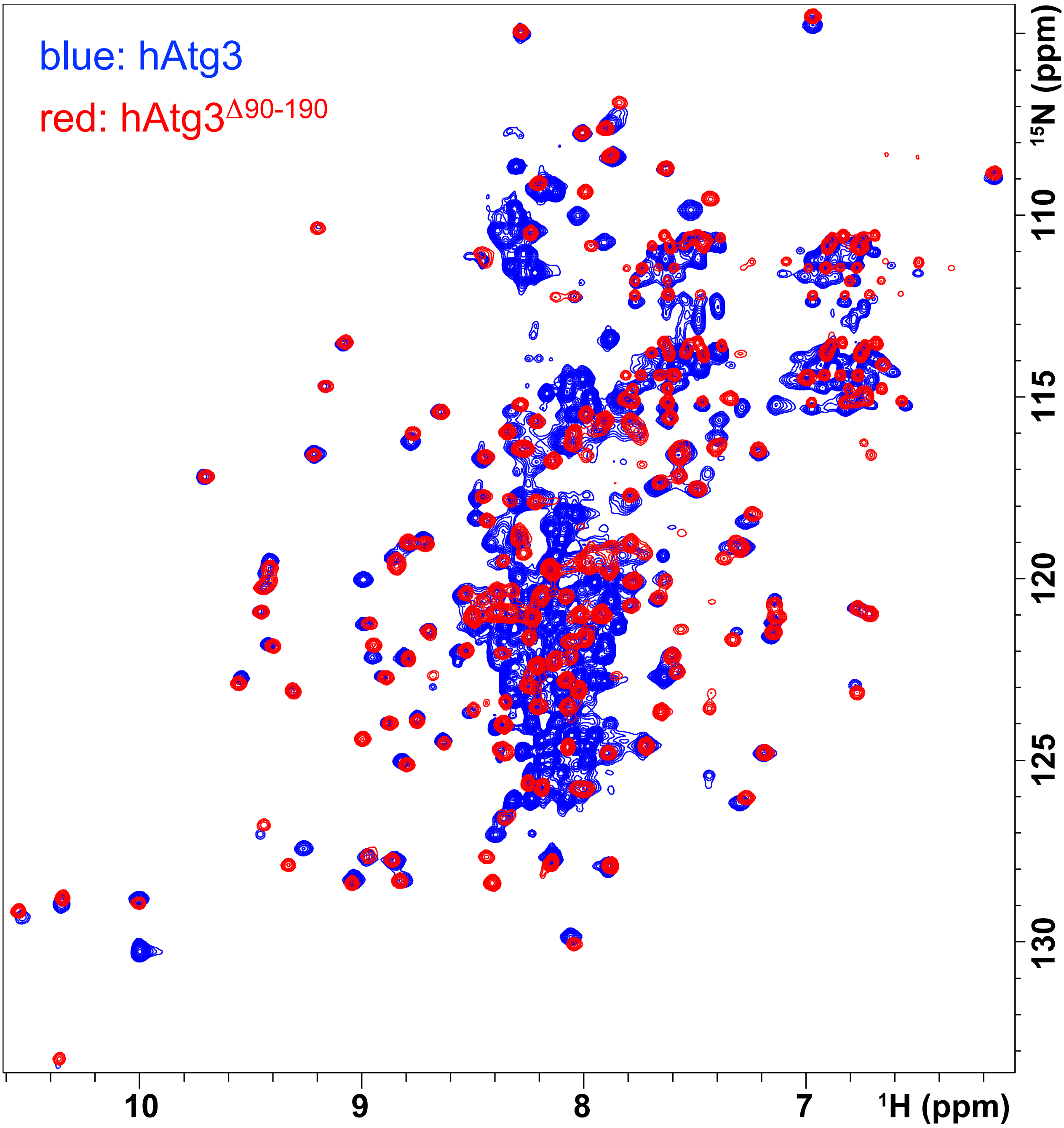

Supplementary Figure 6: Overlay of ^{15}N-^{1}H TROSY spectra of ^{2}H, ^{15}N-labeled hAtg3 (blue) and hAtg3$^{\Delta 90-190}$ (red). Both spectra were acquired on a 600 MHz Bruker spectrometer at 25 °C, pH 6.5.

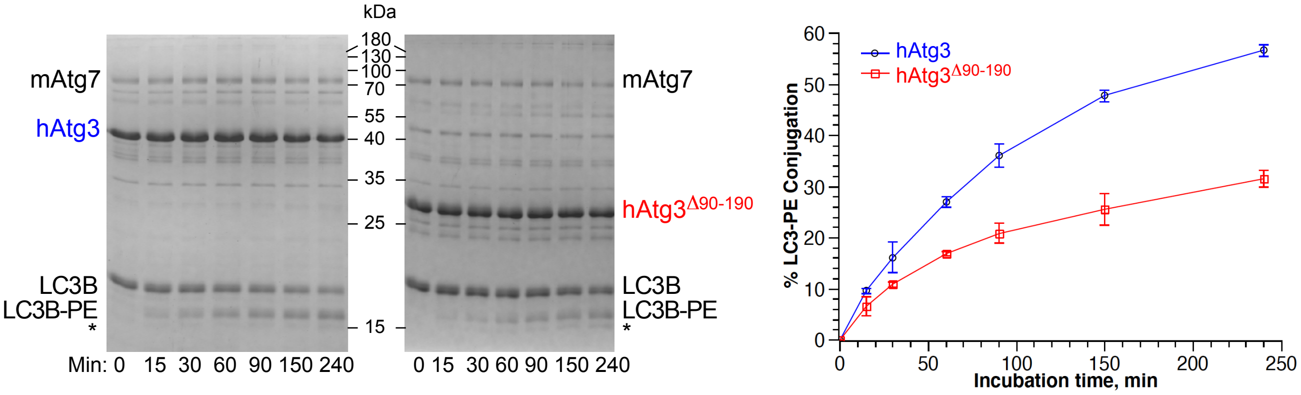

Supplementary Figure 7: hAtg3$^{\Delta 90\text{-}190}$ retains about 50% conjugase activity of the wildtype protein. Gel images and plots of time dependences of LC3B-PE formations for hAtg3 (blue) and hAtg3$^{\Delta 90\text{-}190}$ (red) in *in vitro* conjugation assay. Error bars indicate SD from three independent experiments (n=3). 5 μM hAtg3 or hAtg3$^{\Delta 90\text{-}190}$ was incubated with 1 μM mAtg7 (mouse Atg7) and 5 μM LC3B for 1 hr for the formation of the LC3B-hAtg3 or LC3B-hAtg3$^{\Delta 90\text{-}190}$ intermediates before the addition of liposomes at 0 min.

* a small amount degradation of LC3B in the presence of ATP.

a

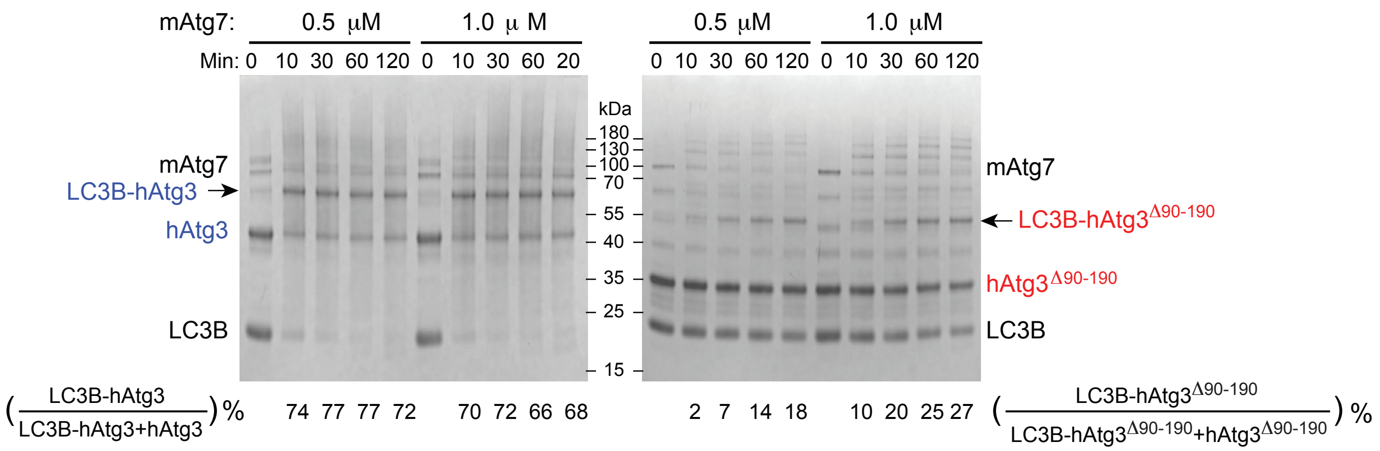

b

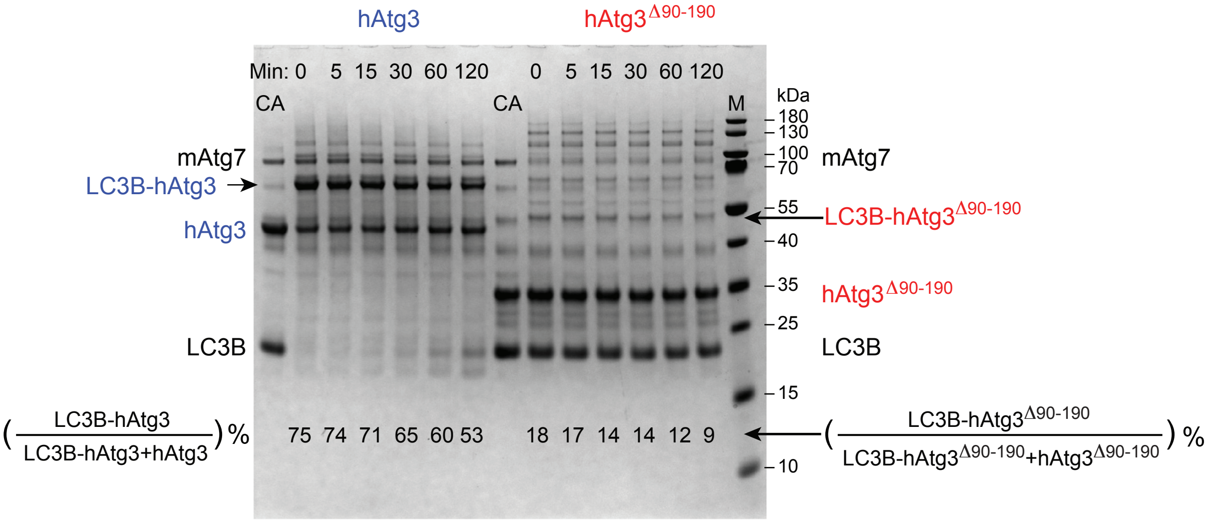

Supplementary Figure 8

c

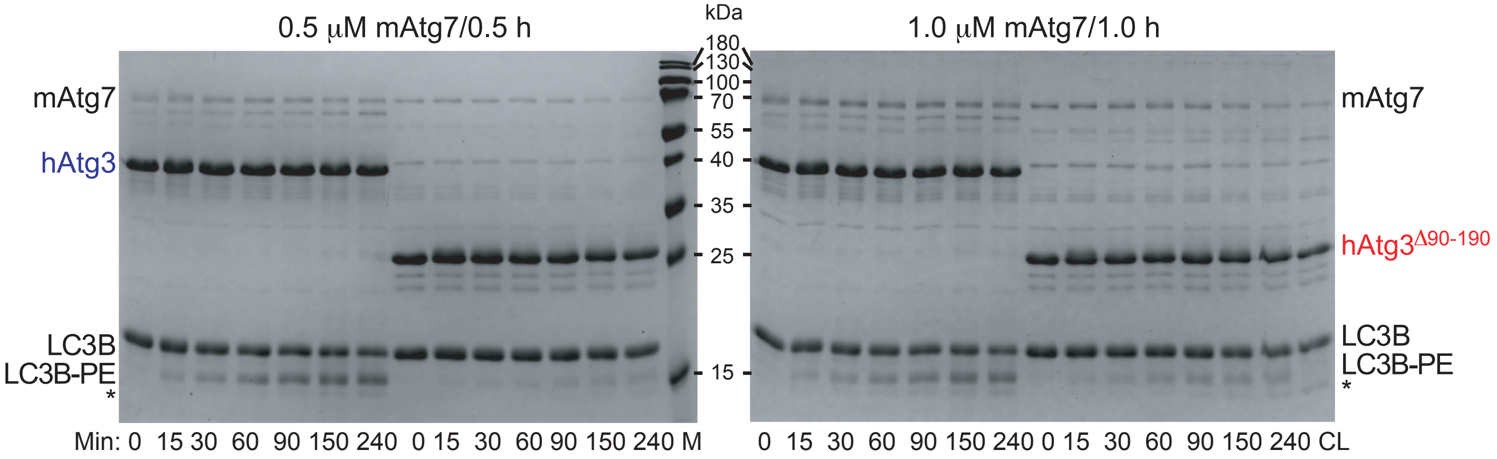

d

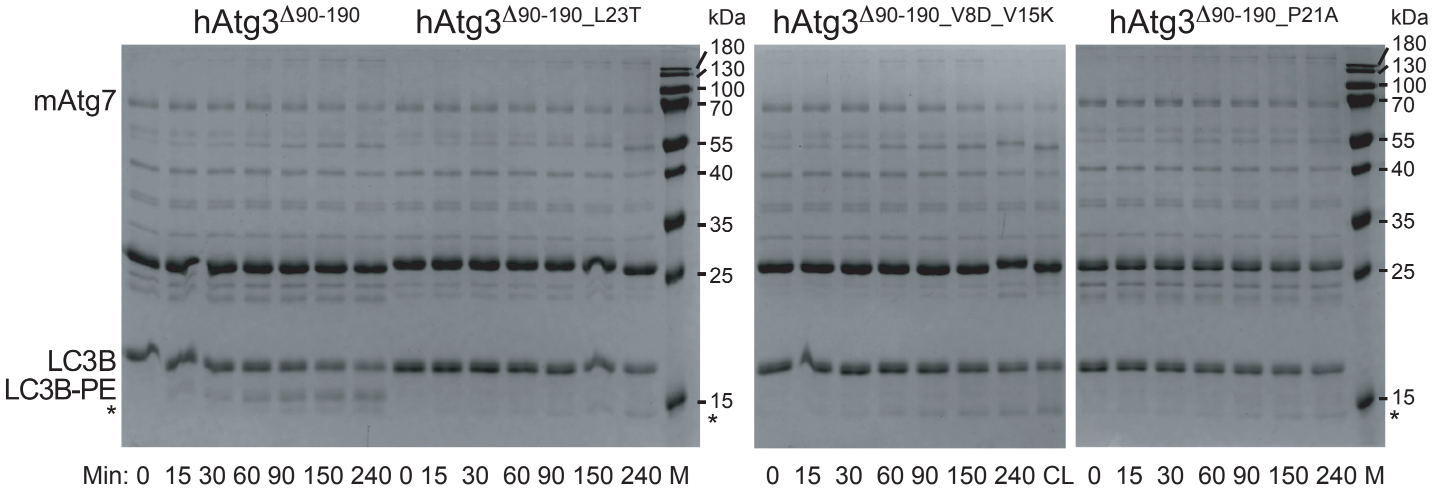

Supplementary Figure 8

Supplementary Figure 8: Intermediate formation and stability for hAtg3 and its mutants.

(**a**) Formations of LC3B-hAtg3 (blue) and LC3B-hAtg3$^{\Delta 90-190}$ (red) in *in vitro* conjugation assay without liposomes. The percentage of intermediates formed is determined by the amount of intermediates divided by the sum of intermediates and hAtg3 (or hAtg3$^{\Delta 90-190}$) and is noted for each time point. Intermediates are detected on a NuPAGE 10% Bis-Tris gel (binding of small amount of LC3B to hAtg3 or hAtg3$^{\Delta 90-190}$ is detected at 0 min before the addition of ATP). While LC3B-hAtg3 formation is very fast at both mAtg7 concentrations, LC3B-hAtg3$^{\Delta 90-190}$ formation is slow and depends on the mAtg7 concentration.

(**b**) Stability of LC3B-hAtg3 and LC3B-hAtg3$^{\Delta 90-190}$ in aqueous solution. The percentage of remaining intermediates is determined by the amount of intermediates divided by the sum of intermediates and hAtg3 (or hAtg3$^{\Delta 90-190}$) and is noted for each time point. 5.0 μM hAtg3 or hAtg3$^{\Delta 90-190}$ was mixed with 5 μM LC3B, 1.0 μM mAtg7, 0.1 mM $MgCl_2$ in a buffer of 37.6 μL. 4.7 μL were removed for a control (CA), and then 1 mM ATP was added and the reaction was allowed to proceed for 1 hr at 37 °C for intermediate formation. 4.75 μL were removed for the first time point (0 min) and 5 mM EDTA was then added to quench the reaction.

(**c**) The conjugation of LC3B-PE for the hAtg3$^{\Delta 90-190}$, but not hAtg3, can be improved by increasing the mAtg7 concentration in the reaction buffer and incubation time for the formation of LC3B-hAtg3$^{\Delta 90-190}$. Gel images of LC3B-PE formation for hAtg3 and hAtg3$^{\Delta 90-190}$ after pre-incubation with 0.5 μM mAtg7 for 0.5 hr, and with 1 μM mAtg7 for 1 hr for the formation of LC3B-hAtg3 and LC3B-hAtg3$^{\Delta 90-190}$ before the addition of liposomes. CL shows the control without liposome.

(**d**) Gel images of LC3B-PE formations for hAtg3$^{\Delta 90-190}$ and mutants after pre-incubation with 1.0 μM mAtg7 for 1 hr before the addition of liposomes at 0 min.

Experiments in a, b, and d were performed once. Experiment in c was repeated three time.

∗ a small amount degradation of LC3B in the presence of ATP.

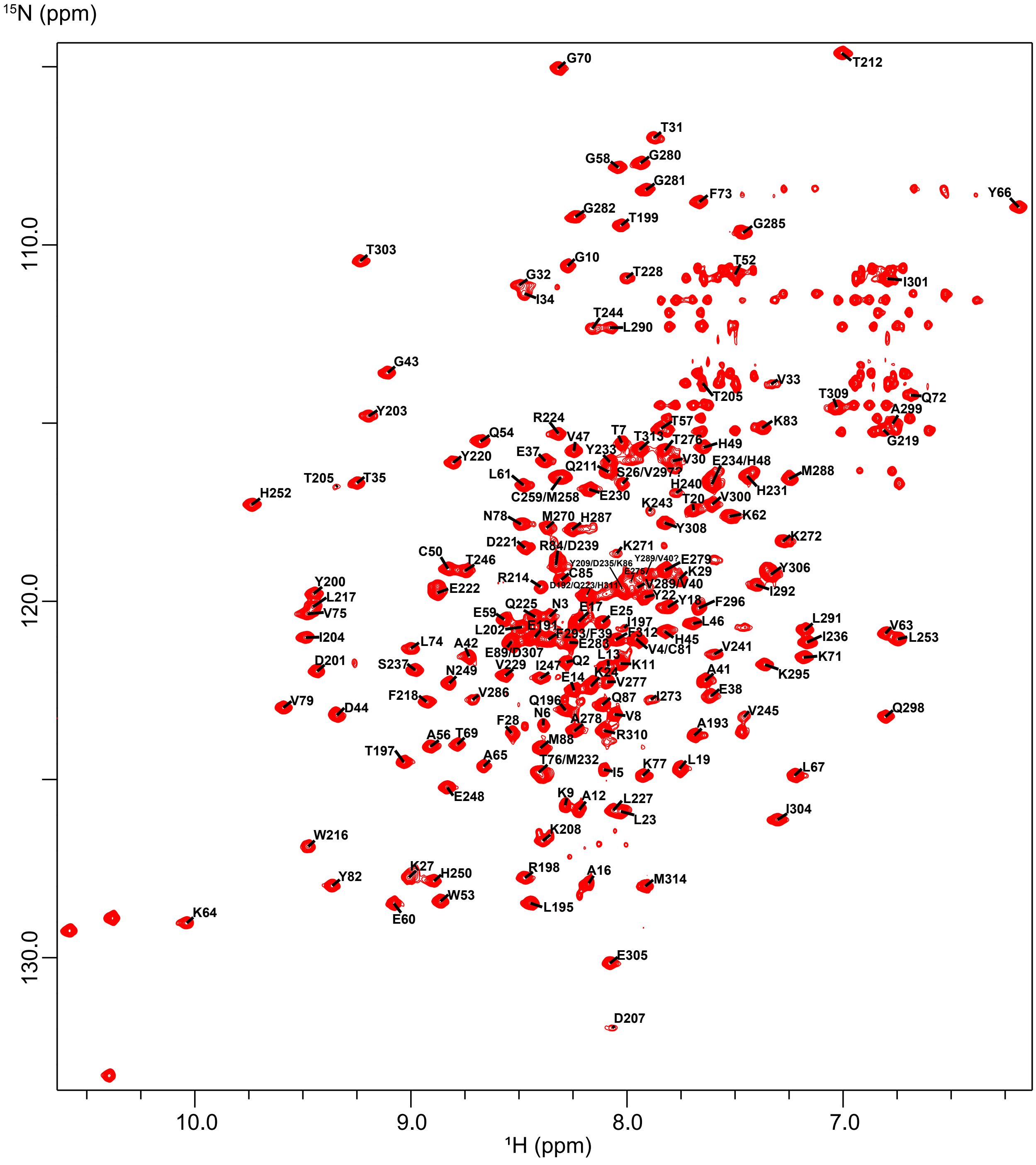

Supplementary Figure 9: ^{15}N-^{1}H TROSY spectrum acquired on a Bruker 600 MHz spectrometer at 25 °C, pH 6.5 for ^{2}H, ^{15}N-labeled hAtg3$^{\Delta 90-190}$ with resonance assignments.

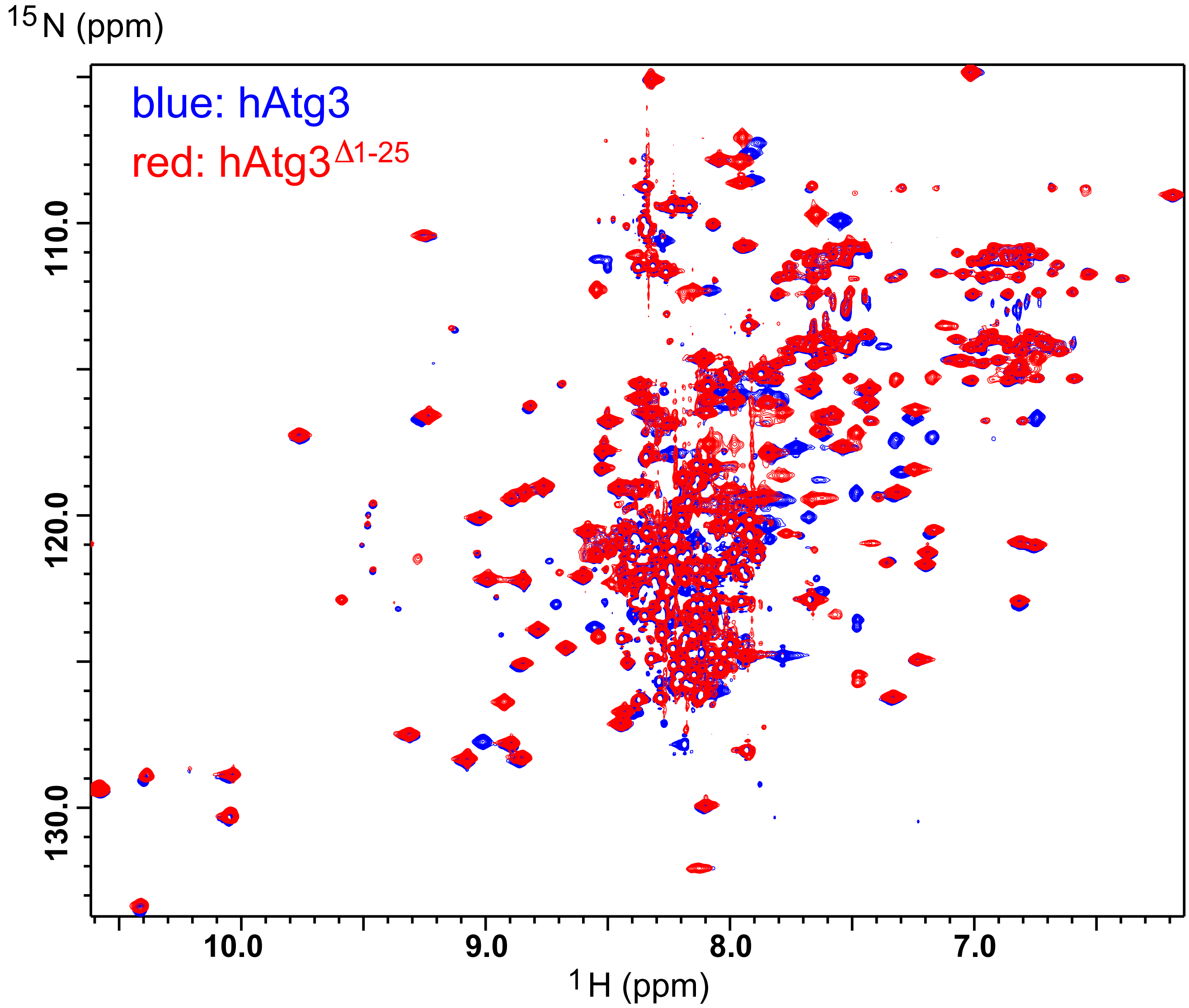

Supplementary Figure 10: Overlay of ^{15}N-^{1}H TROSY spectra of ^{2}H, ^{15}N-labeled hAtg3 (blue) and hAtg3$^{\Delta 1\text{-}25}$ (red). Both spectra were acquired on a 600 MHz Bruker spectrometer at 25 °C, pH 6.5. Deletion of the unstructured region from residues 1 to 25 (hAtg3$^{\Delta 1\text{-}25}$) improves spectral quality for backbone resonance assignments.

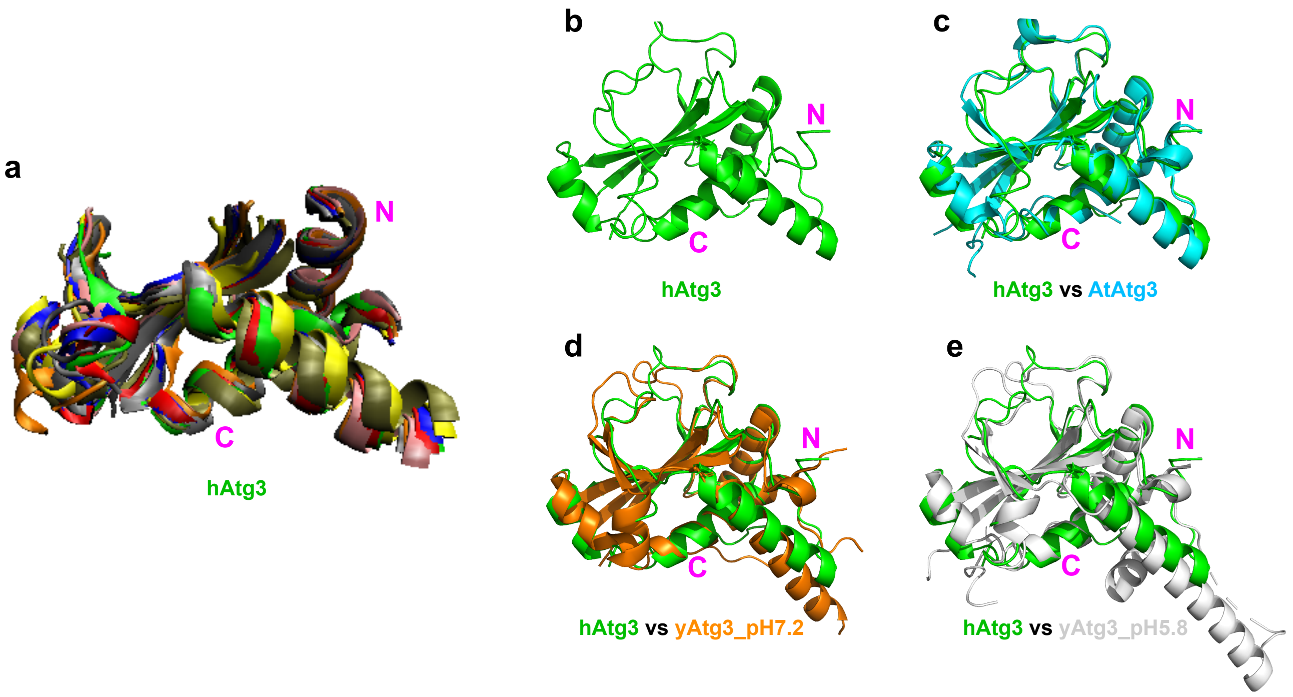

Supplementary Figure 11: Structural models of hAtg3$^{\Delta 1\text{-}25}$ using PONOMA program. (a) Overlay of ten lowest-energy structural models of hAtg3$^{\Delta 1\text{-}25}$ obtained from PONOMA program. The disordered regions are not shown. (b) The lowest-energy structural model of hAtg3$^{\Delta 1\text{-}25}$ obtained from PONOMA program. (c) Overlay of structural models of hAtg3$^{\Delta 1\text{-}25}$ and Atg3$^{\Delta 1\text{-}25}$ from *Arabidopsis thaliana* (PDB: 3VX8). (d) Overlay of structural models of hAtg3$^{\Delta 1\text{-}25}$ and yeast Atg3$^{\Delta 1\text{-}18,\ \Delta 86\text{-}159}$ at pH 7.2 (PDB: 6OJJ). (e) Overlay of structural models of hAtg3$^{\Delta 1\text{-}25}$ with yeast Atg3 at pH 5.8 (PDB: 2DYT). Long disordered regions in hAtg3$^{\Delta 1\text{-}25}$ (residues 86-200) not shown. N and C indicate N- and C- terminals, respectively.

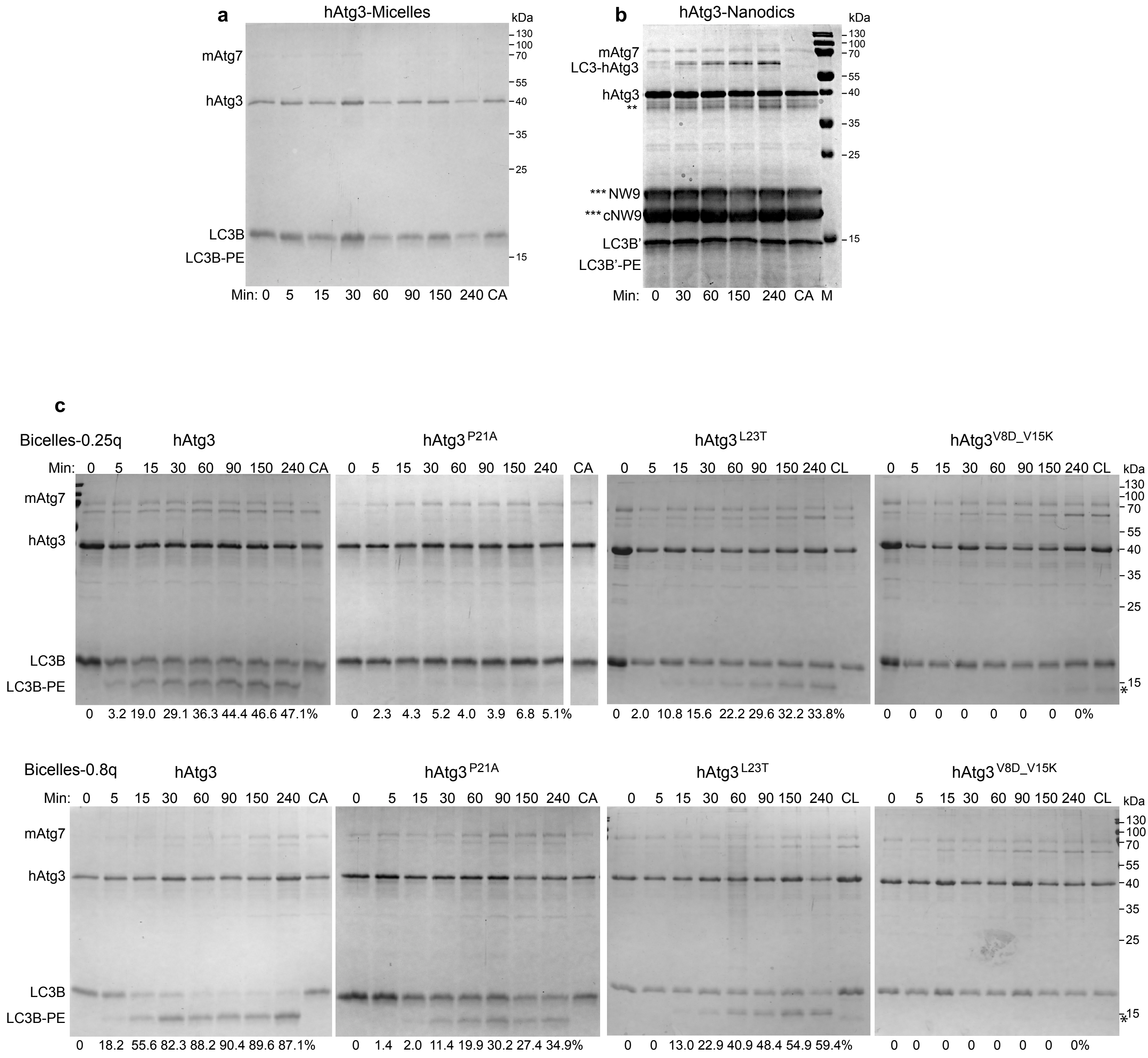

Supplementary Figure 12

Supplementary Figure 12:

SDS-PAGE gel images of LC3B-PE formation in *in vitro* conjugation assay for hAtg3 and its mutants using micelles, nanodics, and bicelles instead of liposomes.

(**a**) micelles (LPC:LPG:LPE=5:2:3, molar ratio).

(**b**) nanodiscs (POPC:POPG:POPE=2:1:2, molar ratio).

(**c**) SDS-PAGE of LC3B-PE formation for hAtg3 and its P21A, L23T, and V8D_V15K mutants using bicelles (DMPC:DMPG:LPE:DHPC=5:2:3:40, q=0.25; DMPC:DMPG:LPE:DHPC=5:2:3:12.5, q=0.8). q is the molar ratio of long chain to short chain lipids. CA represents reaction control without ATP. CL shows the control without lipids. The conjugation percentage of LC3B-PE in bicelles was determined by the amount of LC3B-PE divided by the total LC3B and was noted for each time point. mAtg7 (0.5 μM), hAtg3 and its mutants (5 μM), and LC3B (5 μM) were mixed with ATP to form LC3B-hAtg3 intermediates for 30 min before the additions of bicelles (12% w/v total lipid), nanodiscs (20 μM cNW9), or micelles (100 mM) in reaction buffer at 37 °C. Samples with bicelles and micelles were first pelleted with Ni-beads, and then resuspended in 4x SDS-PAGE sample buffer plus PBS containing 1M imidazole. Samples were boiled at 95 °C for 3 min then subjected to 18% SDS-PAGE gel electrophoresis. LC3B without T7- and His- tags (LC3B') was used with nanodiscs to avoid its overlap with the cNM9 protein.

The conjugation experiments for hAtg3 in bicelles were repeated three times, and experiments for other constructs were performed once.

* a small amount degradation of LC3B in the presence of ATP.

** some contaminants, likely from cNW9.

*** the circularized cNM9 runs faster than the linear NW9 (Nasr, M. L. et al. (2017), Nat. Methods., 14: 49-52).

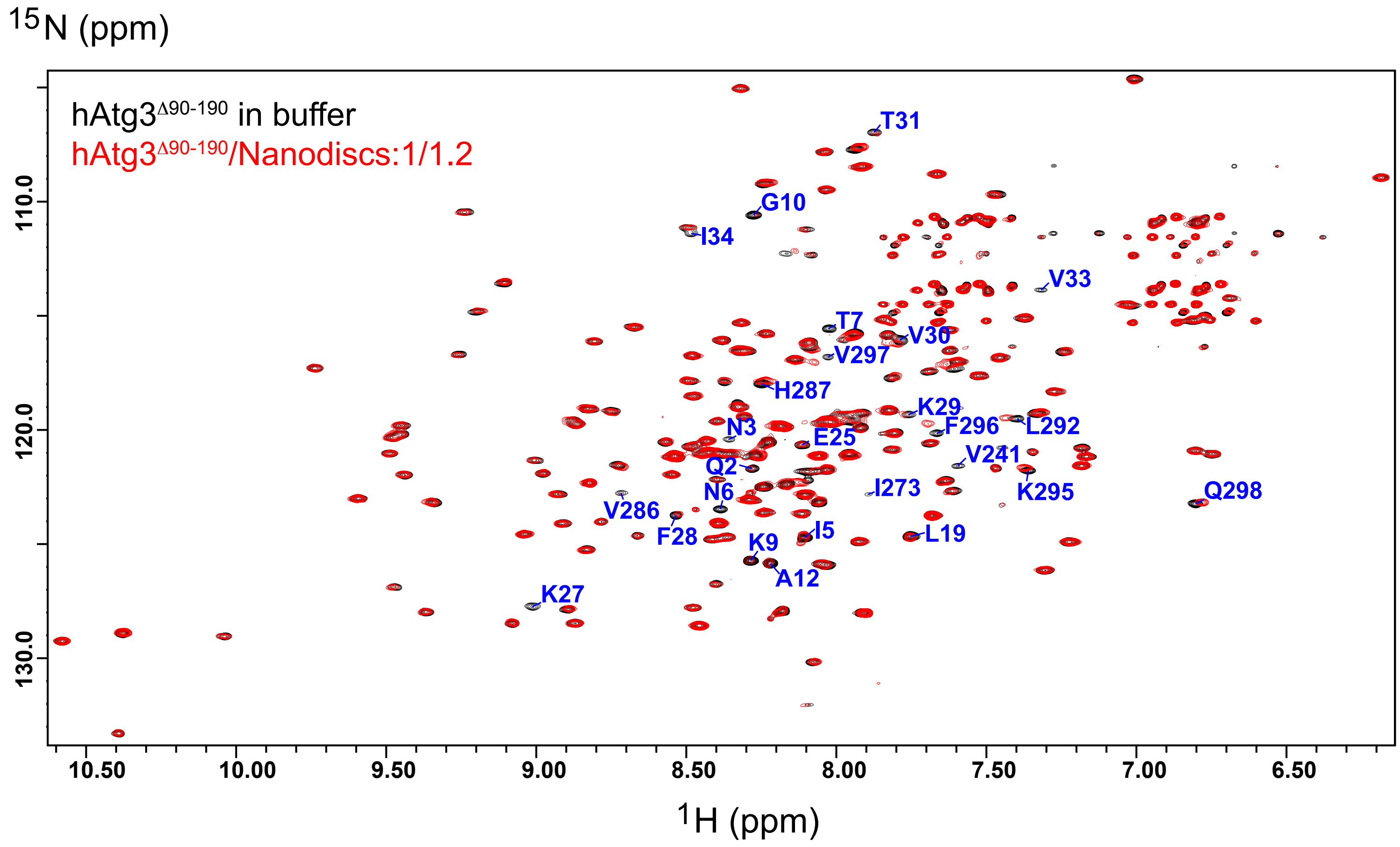

Supplementary Figure 13: ^{15}N-^{1}H TROSY spectra of ^{15}N-labeled hAtg3$^{\Delta 90\text{-}190}$ in aqueous buffer (black) and in nanodiscs (red). The molar ratio of protein to nanodiscs (cNW9) is 1/1.2. Compared to extensive changes seen in the TROSY spectra of hAtg3$^{\Delta 90\text{-}190}$ with and without bicelles shown in Fig.5, perturbations in the presence and absence of nanodiscs are small. Residues with perturbed intensity or chemical shifts are indicated. Both spectra were acquired on a Bruker 600 MHz spectrometer at 25 °C, pH 7.5.

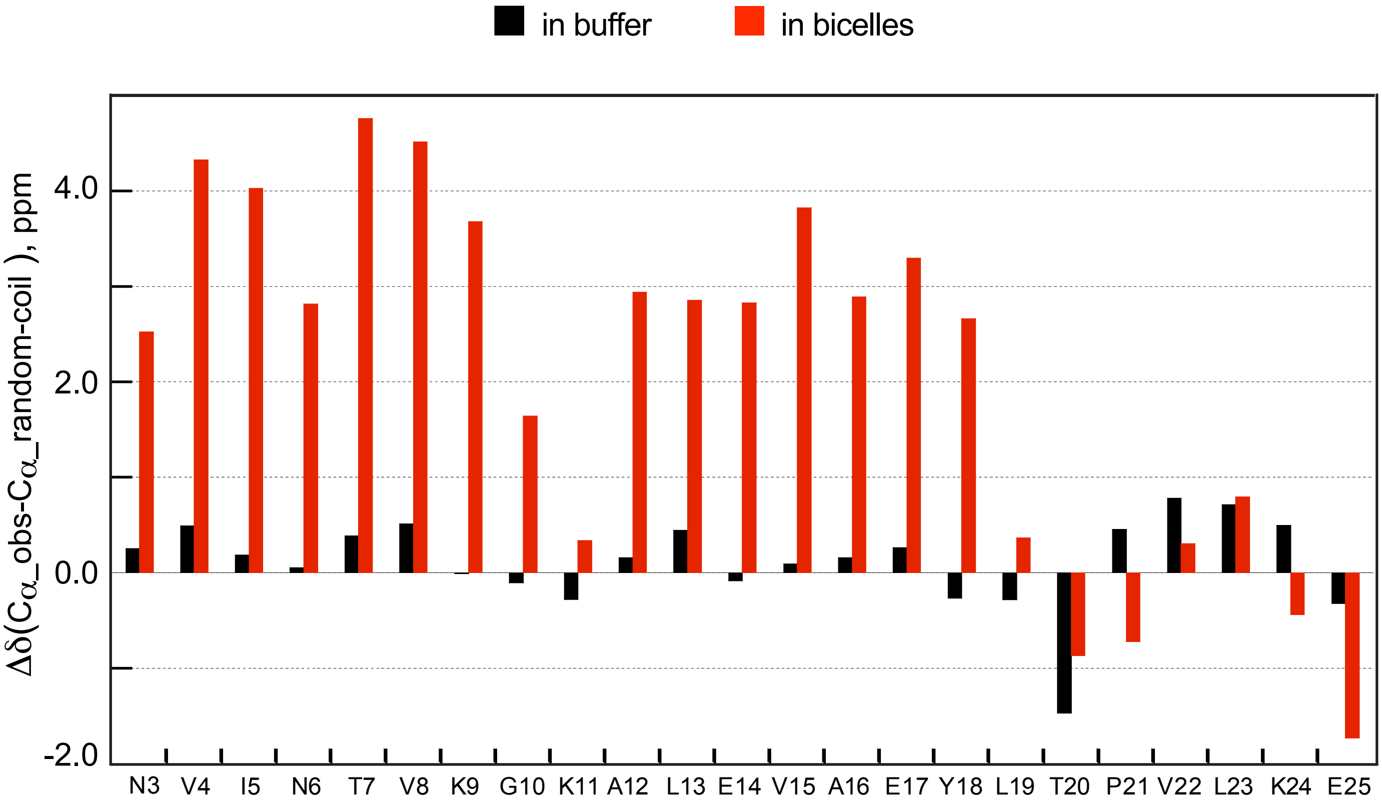

Supplementary Figure 14: $^{13}C_\alpha$ secondary chemical shifts ($\Delta\delta$) indicate that a structural transition from a random coil to an α-helix structure for N-terminal residues upon interaction with bicelles. $^{13}C_\alpha$ secondary chemical shifts for residues 3-25 of hAtg3$^{\Delta 90\text{-}190}$ in aqueous buffer (black) and bicelles (red). C_α_obs is the observed $^{13}C_\alpha$ chemcial shift and C_α_random-coil is the $^{13}C_\alpha$ random coil chemcial shift (Lukin et al., J. Biomol. NMR, (1997), 9: 151-166; Mielke et al., J. Biomol. NMR (2000), 30: 143-153). Deuterium isotope shifts were corrected according to Maltsev et al. J. Biomol. NMR (2012), 54: 181-191.

a

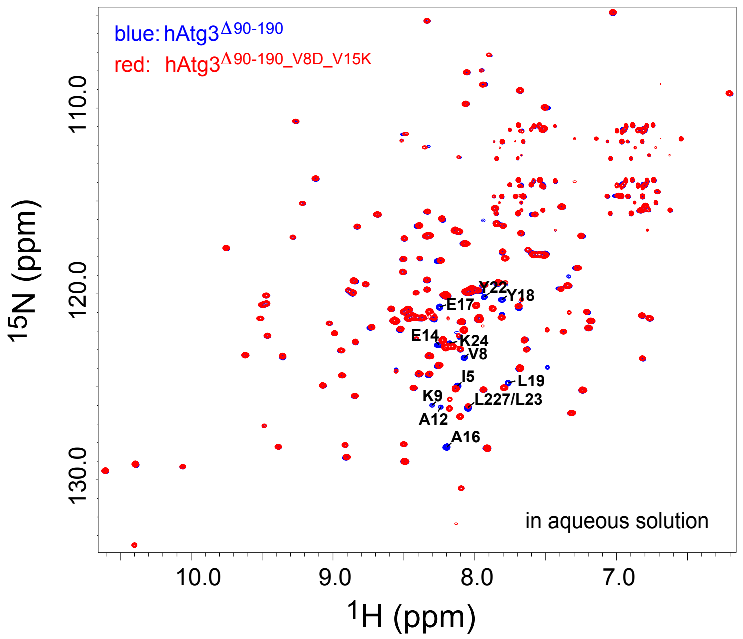

b

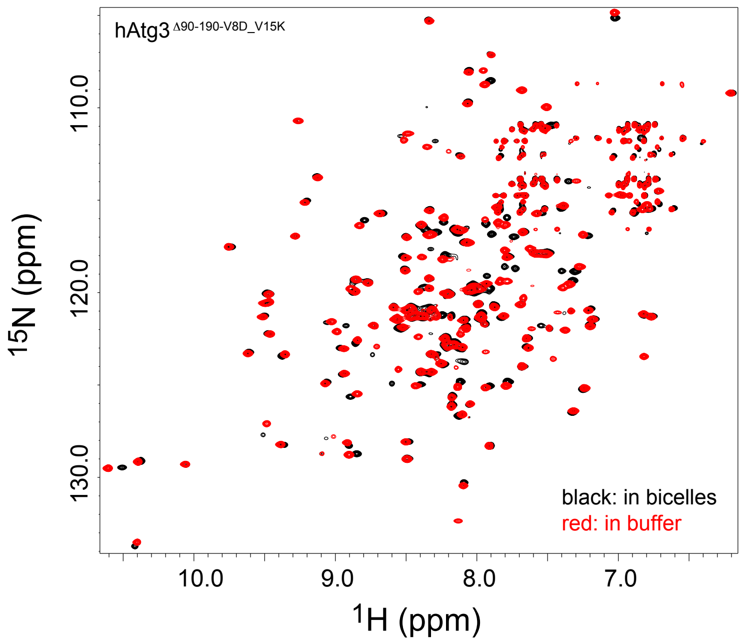

Supplementary Figure 15: V8D_V15K mutation disrupts hAtg3's interaction with membrane.
(a) Overlay of TROSY spectra of ^{15}N-labeled hAtg3$^{\Delta 90-190}$ (blue) and hAtg3$^{\Delta 90-190_V8D_V15K}$ (red) in aqueous solution. The perturbed residues are indicated and are around the mutation site.
(b) Overlay of TROSY spectra of ^{15}N-labeled hAtg3$^{\Delta 90-190_V8D_V15K}$ in aqueous solution (buffer, red) and in bicelles (black). Compared to hAtg3$^{\Delta 90-190}$ shown in Fig. 5, perturbations for the V8D_V15K mutant in the presence of bicelles are much smaller. All spectra were acquired on a Bruker 600 MHz spectrometer at 25 °C, pH 7.5.
