# Supplementary material for: An N-terminal conserved region in human Atg3 couples membrane curvature sensitivity to conjugase activity during autophagy
Source: Nat Commun. 2021 Jan 14;12:374. doi: 10.1038/s41467-020-20607-0 (PMC7809043; doi:10.1038/s41467-020-20607-0)
Supplement: Supplementary file 2 — Peer Review File [file 41467_2020_20607_MOESM2_ESM.pdf]

REVIEWER COMMENTS

Reviewer #1 (Remarks to the Author):

The manuscript from Tyndall et al, uses a series of biochemical and biophysical assays to uncover a novel element in the activation of the autophagy enzyme, ATG3. Previous work had identified an amino-terminal amphipathic helix on ATG3 that contributes to its interactions with membranes and is essential for its ability to conjugate LC3 proteins with the membrane-embedded lipid, phosphatidylethanolamine. Here, Tyndall and colleagues demonstrate that the structure of ATG3 around its active site is altered following membrane binding, and furthermore, that a sequence proximal to the amino-terminal helix is necessary to the transduce the signal from membrane-binding to active-site-rearrangement. Many proteins in autophagy, including ATG3, are proposed to recognize highly curved surfaces and to likely concentrate at the curved rim of the isolation membrane. The work here, that simply binding a curved membrane is not sufficient for activity, but instead that the protein must be forced into a correct and active orientation, will be of interest to work on this protein and to the field of autophagosome formation in general.

The interpretations of their results are, on the whole, convincing, however there are a few areas that require additional work (noted below). Foremost, for many of the key experiments, replicates/error are not presented and as these approaches can have natural variation on the same scale as the data they present, the replicates are essential. These include the reconstitution of ATG3 activity in vitro, where variations in liposomes or protein specific activities can be as dramatic as their apparent signal differences, and the rescue of ATG3 activity in cells, where they currently show only one blot that suggests that the ATG3 proline mutant is less active (though not inactive) in a system where simple changes in expression level have previously been shown to inhibit activity. These points and several others are detailed below and should be addressed.

1) Why focus on the proline mutation? The rationale around why the authors zeroed in on this mutation is not well developed in the text and would be helpful for following their plan. Likewise, it is not clear why only the in vitro results for the proline mutation are shown with replicates and significance in Figure 3. These experiments are fairly trivial and all should be quantified. What is the CD result of the proline mutation, one could easily imagine a fairly dramatic change in helicity associated with altering a key proline. More broadly, the authors discuss each of the mutants in Fig. 3 in the text, proposing that the yeast and human proteins have distinct but conserved membrane binding motifs ahead of the key transduction motif, but all of this is speculation based on the enzyme activity (of lipidation). However the authors have developed a very nice intrinsic tryptophan fluorescence assay to follow membrane binding directly that can and should be used for each of these mutations to establish whether they are merely uncoupling membrane binding from activity or alternatively, disrupting membrane binding itself. Finally, they note in the text that all of the mutants are capable of forming the thioester intermediate, but do not show it. Again, that is a very simple experiment (just a blot of the assays they have already run) and should be shown.

2) In vivo rescues – Figure 4 needs to have replicates as independent lentiviral transductions. As there is some lipidation in the proline-mutant, the degree of reduced activity could reflect the mutation itself (as authors suggest) or a variance in autophagy activity due to apparently higher expression of this mutant. Several proteins in the lipidation cascade have been shown to inhibit activity in cells when overexpressed (or underexpressed) significantly. Furthermore, could the authors comment on the "flux" they observe? There is no apparent increase in total LC3-II following Baf except under starvation, and even then it does not appear to accumulate beyond what was already present in the unstressed no-Baf condition. These values should be quantified from replicates and included in the figure. Alternatively, another flux substrate such as p62 could be used.

3) DATA quality problems: In Figure 3, the top panel in both 3a and 3b appears to be an identical gel. Were all of these experiments conducted on the same day from the same samples? As above, more information on replicates is necessary. In Figure S7, the gel for p21A-0.25Q appears to have the last lane simply pasted on. If this is data from another gel (or from a separate region on the same gel), this piece should be shown as actually separate rather than connected to other

samples.
4) Why do bicelles work when other membrane mimics do not? The authors write that "The observation that hAtg3 is more active in larger bicelles (Fig. S7d) further supports the notion that the protein interacts with the planar surfaces instead of the edges of bicelles that have a curvature radius of approximately 5 nm." This explanation is not obvious to me, as the larger bicelles will also have more curved surface area. If the binding is actually via the curved edge, would this impact the way the authors interpret their story, as a 5 nm radius of curvature is probably not representative of the membranes in a cellular environment?
5) Impact of the deletion mutant (delta 90-190) – The authors are able to make an impressively large deletion in ATG3 without massively impacting its activity, and thus are able to use this deletion for subsequent structure analysis. However, in measuring the activity, they speculate that the reduced lipidation driven by this mutant reflects a reduced rate of ATG3-LC3 conjugate formation due to loss of ATG7 binding. That would suggest this first coupling event is rate-limiting, but the data in SFig4 b and c are hard to square with that interpretation. As in other places, it would be helpful if replicates were shown. Is SFig4b representative, in which case the impact on lipidation is fairly mild, however, in Sfig4C they observe almost no ATG3-LC3 conjugate formation suggesting the enzyme is nearly dead. I wonder if an alternative interpretation is that loss of the loop destabilizes the ATG3-LC3 conjugate and thus it does not survive the sample preparation ahead of running the gel.
6) In vitro conjugation in methods – "The in vitro conjugation was conducted similarly to previous descriptions87,142." There are only 34 references in this paper, please double-check the references in the methods section.
7) Suggestions for clarity -- The nomenclature "NTconv" intended to be short for conserved N-terminus might be clearer as NTcon or NTcons. Note on pg 10, "Fig. S6d" should actually read "Fig. S7d".

Reviewer #2 (Remarks to the Author):

Autophagy is a membrane traffic pathway, in which different cellular materials are sequestered within autophagosomes and transported to lysosomes for degradation. Ubiquitin-like Atg8-family proteins (LC3s and GABARAPs in mammals) are anchored to nascent autophagosomal membranes via their lipidation (conjugation to phosphatidylethanolamine (PE)), and play multiple roles, including autophagosome formation and cargo recognition. Lipidation of Atg8-family proteins is mediated by the ubiquitin-like conjugation reaction, in which Atg3 serves as an E2 enzyme that conjugate the proteins to PE. Previous studies have revealed that the N-terminal region of Atg3 contains an amphipathic helix (AH), which is responsible for membrane binding of this enzyme and thus is essential for its conjugase activity. In this study, Tyndall et al. found that the N-terminal region of Atg3 additionally contains a short conserved segment C-terminal to the AH. This segment was proven to be required for LC3 lipidation but seemed not to be involved in membrane binding of Atg3. NMR analyses suggested that Pro21 in this segment is required for conformational changes in the catalytic core domain of Atg3 that are induced by its membrane (bicelle) binding. Taken together, the authors proposed a model that this conserved segment acts to transmit a signal of membrane binding in the AH to the core domain to stimulate Atg3 conjugase activity. However, several major issues remain to be addressed to allow the authors to draw this conclusion.

Major comments:
(1) The authors should perform the experiments shown in Figs. 4, 6, S3, and S7, and additional experiments requested below for the L23T mutant to know the role for this mammal specific residue.

(2) Membrane binding assay based on changes in tryptophan fluorescence should include a previously-described AH mutant defective in membrane binding to show that the changes were indeed resulted from the membrane binding of Atg3 in the AH. In addition, it would be better if more direct methods such as liposome flotation assay will be performed. CD spectrometry should also be performed for peptides containing the P21A and L23T mutations to confirm that the membrane association property of the mutant proteins was not affected by these mutations.

(3) Confirming thioester intermediate formation of the Atg3 mutants is critical and therefore should be presented in the manuscript.

(4) Since in vivo experiments are not clear enough, the quantification will be required. Immunoblot of p62 will also be helpful to evaluate autophagic flux.

(5) The authors should perform CD spectrometry and membrane binding assay using bicelles for wild-type and mutant peptides and proteins to show that the results of these analyses using liposomes can be discussed along with structural information obtained by NMR, in which bicelles were used.

(6) The authors should clearly state whether the P21A mutant did not cause a conformational change upon the addition of bicelles or caused a conformational change different from the wild-type protein.

(7) In the present manuscript, there is no evidence supporting the model that the conserved segment works downstream of membrane binding in the AH to cause conformational changes in the core domain to increase Atg3 conjugase activity. The authors have to show that a mutation in the AH that impairs the membrane binding of Atg3 cancels the conformational changes caused by bicelles as with the case for the P21A mutation.

Minor comments:
(1) Page 3, line 3: The phagophore is a cup-shaped "single" membrane vesicle.
(2) Page 3, line 12: Atg4 removes not the last residue but several residues from the C-terminal region of LC3.
(3) In some parts in the text, the authors use the words "temporally" or "spatio-temporal", but the focus of the present study is not related to the temporal regulation of LC3 lipidation or autophagosome biogenesis.
(4) The relationship between human and yeast proteins should be described as "homologs or orthologs" but not "isoforms".
(5) Page 10, line 12: "Fig. S6d" should be "Fig. S7d".
(6) It may be better if the positions of Y220, S237, E248, and H252 are also shown in Fig. 5c.

Reviewer #3 (Remarks to the Author):

The manuscript reports the identification of a - hitherto overlooked - conserved sequence motif in the N-terminal region of the autophagy-related protein Atg3, which plays an essential role in autophagosome formation. By investigating several yeast-human chimeric constructs via in vitro and in vivo assays the authors show that this motif is required for efficient conjugation of LC3 family proteins to phospholipids at the tip of the autophagic membrane. An extensive set of NMR spectroscopic experiments suggests that this motif couples the coil-to-helix transition of the N-terminal membrane curvature sensor to a conformational change near the active center in a manner reminiscent of allosteric regulation.

The paper is based on an extensive body of convincing experimental data. The results are of sufficient novelty and of considerable biological interest, and in the discussion the authors point out (convincingly again) that the study opens up follow-up studies geared at understanding the exact mechanism of this conformational switch. Unfortunately, the level of detail given in the present manuscript does not always live up to the standard of allowing an expert reader to fully understand and reproduce the experiments. I would therefore recommend publication of this manuscript in Nature Communications after major revisions, as described below.

While this interesting manuscript is written in a very readable style overall and has the potential to become a real pleasure to read in a revised version, I found the presentation of the sequence alignments a little difficult to digest. It is split between the introduction, results section, Figs. 1, 2,

and S1. The font size in the figures is rather small and hard to read. The color codes used in the figures are different from figure to figure, largely unexplained, and not particularly intuitive. The order of species in the sequence alignments also appears pretty random to me. All the necessary information can be found somewhere in these sections, but I think the presentation of this important part of the manuscript could really benefit from a careful make-over towards a clearer and more unified presentation.

Maybe my biggest concern is the general lack of detail provided, especially in the figure legends. There are more unexplained color codes, abbreviations, symbols, units etc. in this manuscript – especially in the figure legends – than I can list here. In the case of Fig. 4, the legend is so sparse that I could not even understand the figure any more. The NMR spectra are presented without any details, not even the magnetic field strength used, even though it is important for the peak position in TROSY spectra. And the structural models and overlays are small and unlabeled, not even the N- and C-termini are marked, which makes the orientation very cumbersome. And there are several sentences in the manuscript with a missing or unclear (literature or figure) reference.

The authors present sequence-specific NMR resonance assignments and even use these chemical shifts for homology modeling using PONOMA, but they have not made the underlying chemical shifts publicly available. It is standard practice in the community and (as far as I know) a requirement of the editorial policy of Nature journals to deposit chemical shift assignments with the BMRB before publication. It might also be worthwhile to make the PONOMA results available for download, but this is optional.

Individual comments:
* Abstract: "structurally distinct" is not entirely clear here.
* In humans, Atg8 is not a single protein but a family of at least 6 proteins divided into two sub-families (GABARAP-like and LC3A/B/C). The expression "LC3" used throughout this manuscript is therefore highly confusing. Please clarify exactly which protein(s) each instance of "LC3" refers to. From what I understand, the authors mostly use LC3B, which raises the question whether the results could be different for other members of the LC3/GABARAP family. Please discuss.
* "we synthesized two peptides that correspond to the NT regions of hAtg3 and yAtg3 (Fig. 1a)" – I cannot see these constructs in Fig. 1a.
* Not just the figure legend to Fig. 4 is a little sparse, so is the explanation in the main text. I do not think the readers should be forced to read ref. 20 to be able to comprehend the present manuscript.
* "their resonances are not observed as a result of intermediate exchange" - Is there any hard evidence that this can be attributed to chemical exchange, or is this just a (plausible) hypothesis? Please clarify.
* "show good convergence […] (Fig. S6)." - I cannot see ten structural models in Fig. S6 and therefore no RMSD and no convergence.
* "comparable to that in liposomes (Fig. S6d)" – could this be a reference to Fig. S7d?
* "Atg3 is more active in larger bicelles (Fig. S7d)" – I do not find this straightforward to see in Fig. S7d.
* From what I understand (difficult to say due to the lack of detail in the methods section), the chemical shifts of hAtg3 bound to bicelles had to be determined from triple-resonance experiments, which suggests to me that 13C chemical shifts are available as well. 13C chemical shifts are highly sensitive to any changes in secondary structure. Are there any detectable changes in regular secondary structure upon membrane binding, or are the "structural rearrangements" more subtle?
* "albeit with decreased Atg12-Atg5 conjugation" – this sentence was not clear to me.
* "similar to the configuration seen in the active site" (article missing)
* Legend to Fig. 3: Not sure "respectively" is used correctly here.
* "was exchanged to a thrombin cleavage buffer" – how was the buffer exchange performed?
* "BME" – beta-mercaptoethanol?
* I don't think there are any references numbered 87 and 142.
* "reaction buffer (50 mM HEPES, 150 mM NaCl" (typo)
* "Gels were destained" - or is there such a thing as a detention center for gels? ;-)
* "with a 0.5 mm bandpass" – mm or nm?
* "hAtg3yMin" – which construct is this?

* The NMR methods section is far, far, far too sparse on details and references.
* "For the stability of samples with 12% bicelles the pH was kept at 7.5." - unclear
* Fig. 5a is a bit on the small side.
* Fig. 6c: "aqueous" (typo)
* Fig. S2: "require the presence of negatively charged lipids (PGs or PIs)" – I cannot see any control without PGs or PIs? Also, exactly which lipids were used?
* Fig. S3: Why does the Trp fluorescence decrease upon liposome binding? Naively, I would have expected it to become more protected from water and hence to increase...
* Fig. S4b: The figure resolution looks a bit pixelated. Also, "Atg7m" is not explained, I think.
* Fig. S7c is definitely too small.
* Fig. S8: How was the secondary chemical shift calculated (i. e., what is Ciso)? (Reference necessary.) Upon closer inspection, there is a systematic downfield shift of about 1.5 ppm even in buffer, which exceeds the CSI cutoff of +0.8 ppm for helical conformation (Wishart & Sykes, Meth. Enzymol. 239, 363-392), suggesting that there is already significant helical propensity. And the downfield shift of 4..5 ppm with bicelles is larger than expected ("As a general rule, 13Ca shifts experience a downfield shift of about 2.5 ppm in helices", Wishart & Case, Meth. Enzymol. 338, 3-34). Please double-check the referencing. Was this a deuterated sample? If so, did you correct for isotope effects?

Reviewer #4 (Remarks to the Author):

The manuscript by Tyndall et al. entitled «An N-terminal conserved region in human Atg3 couples membrane curvature sensitivity to conjugase activity during autophagy» shed a light on a very interesting and important topic – how the activity of autophagy E2 ligase Atg3 (expressed in conjugation of human Atg8 homologs LC3/GABARAP proteins to phosphatidylethanolamine) correlates with the specific membrane curvature at the edges of growing autophagocome. N-terminal evolutionary conserved region of Atg3 proteins was recently identified to form an amphipathic helix (AH), which senses membrane curvature and couples Atg3 conjugation activity to the growing phagophore. However, the mechanism of this correlation remained unknown. The authors made a big step toward to understanding of the relationship between functional and structural interplay within the Atg3 family proteins. They analyzed the N-terminal region (NT) of Atg3 proteins (residues 1-26 in human Atg3) and found that residues 3-19 are involved in the AH formation. Residues 1-16 are rather variable (NTVar), while residues 17-26 are highly conserved NTConv). The authors unambiguously demonstrated that NT adopts a helical conformation exclusively in presence of the small-size liposomes, being unstructured in aqueous solution or in presence of larger size liposomes. By in-vitro conjugation assays and by ability of several Atg3 chimeric constructs and mutants to rescue LC3 lipidation in Atg3-/- mouse embryonic fibroblasts (in-vivo), authors demonstrated that the Atg3 activity lost is predominantly due to alteration of the NTConv. In order to understand molecular basis of the observed functional significance of NTConv, they undertook a series of NMR experiments to visualize chemical shift perturbations (SCP) in presence of curved membrane-mimicking bicelles (here I should mention that the NMR work presented here - including the Atg3 constructs optimization, screening of membrane-mimicking components and assignment of resonances in the media - is extremely elegant and informative). Results of these experiments indicate that in presence of the corresponding bicelles i) Atg3 residues 3-19 form a helical structure; ii) significant CSP are observed at the C-terminally located catalytic core of Atg3, indicating structural rearrangements of the core. Comparison of the CSP for Atg3 and its P21A mutant (the mutant with almost complete abolished LC3 lipidation activity in-vitro and in-vivo) reveal that the P21A mutation affects both NT and catalytic core structures, disrupting productive interaction between both and inactivating Atg3.

In summary, this study provides new and important insights on the functional correlation between the N-terminal membrane-curvature sensing part of Atg3 and its C-terminal catalytically active part. Experimental designs are solid, and the results are clear and sufficient to draw the conclusions the authors made in the manuscript. Almost all necessary controls were performed and obtained results were consistently interpreted. Therefore, I strongly recommend this research paper for publication in "Nature Communications" after minor revision.

I have just a few concerns:

The hAtg3 optimized construct used for NMR experiments (hAtg3Δ90-190) was approved to be able to lipidate LC3 in-vitro (Fig S4b) but its inactive mutant - hAtg3Δ90-190,P21A - was not tested in-vitro, gaining the lack of this negative control.

Additionally, the both deletion constructs were not tested in-vivo (LC3 lipidation level and autophagy flux for hAtg3Δ90-190 and hAtg3Δ90-190,P21A in the Atg3-/- MEFs, the rescuing experiments). I believe that these experiments are necessary as kind of positive controls which bring together the structural, biochemical and cellular parts of the story. In that case the results and conclusions made by authors will be more convincing.

Minor points:
1. At Figure 1b the sequences might be centered on the EYLTP sequence for better understanding.
2. Figure S7c is too small to be clearly seen, authors should increase either size of the NMR plot or image resolution.

Response to the Reviewers

We highly appreciate the reviewers for their encouraging and constructive comments. We considered them at length and endeavored to provide the requested experimental data and revised the manuscript accordingly. As a result, we think the manuscript has become much stronger.

Reviewer #1 (Remarks to the Author):

The manuscript from Tyndall et al, uses a series of biochemical and biophysical assays to uncover a novel element in the activation of the autophagy enzyme, ATG3. Previous work had identified an amino-terminal amphipathic helix on ATG3 that contributes to its interactions with membranes and is essential for its ability to conjugate LC3 proteins with the membrane-embedded lipid, phosphatidylethanolamine. Here, Tyndall and colleagues demonstrate that the structure of ATG3 around its active site is altered following membrane binding, and furthermore, that a sequence proximal to the amino-terminal helix is necessary to the transduce the signal from membrane-binding to active-site-rearrangement. Many proteins in autophagy, including ATG3, are proposed to recognize highly curved surfaces and to likely concentrate at the curved rim of the isolation membrane. The work here, that simply binding a curved membrane is not sufficient for activity, but instead that the protein must be forced into a correct and active orientation, will be of interest to work on this protein and to the field of autophagosome formation in general.

The interpretations of their results are, on the whole, convincing, however there are a few areas that require additional work (noted below). Foremost, for many of the key experiments, replicates/error are not presented and as these approaches can have natural variation on the same scale as the data they present, the replicates are essential. These include the reconstitution of ATG3 activity in vitro, where variations in liposomes or protein specific activities can be as dramatic as their apparent signal differences, and the rescue of ATG3 activity in cells, where they currently show only one blot that suggests that the ATG3 proline mutant is less active (though not inactive) in a system where simple changes in expression level have previously been shown to inhibit activity. These points and several others are detailed below and should be addressed.

Author reply: We apologize for not including these data in our first submission, and have included the experimental data and replicates on membrane binding, formation of thoiester intermediate, *in vitro* reconstitution assay, and *in vivo* cellular assay for hAtg3 and its variants in Fig. S3, S4, S5, and Fig. 4

1) Why focus on the proline mutation? The rationale around why the authors zeroed in on this mutation is not well developed in the text and would be helpful for following their plan. Likewise, it is not clear why only the in vitro results for the proline mutation are shown with replicates and significance in Figure 3. These experiments are fairly trivial and all should be quantified. What is the CD result of the proline mutation, one could easily imagine a fairly dramatic change in helicity associated with altering a key proline. More broadly, the authors discuss each of the mutants in Fig. 3 in the text, proposing that the yeast and human proteins have distinct but conserved membrane binding motifs ahead of the key transduction motif, but all of this is speculation based on the enzyme activity (of lipidation). However the authors have developed a very nice intrinsic tryptophan fluorescence assay to follow membrane binding directly that can and should be used for each of these mutations to establish whether they are merely uncoupling membrane binding from activity or alternatively, disrupting membrane binding itself. Finally, they

note in the text that all of the mutants are capable of forming the thioester intermediate, but do not show it. Again, that is a very simple experiment (just a blot of the assays they have already run) and should be shown.

Author reply: We chose to replace an invariant proline with an alanine because proline residues often play key structural and functional roles. Our previous work has shown that a proline in SpoVM is critical for its membrane curvature-sensing (Gill et al. 2015, PNAS, 112: E1908-E1915). Along with this added rationale in the revised text, we have included the CD data to show that the NT point mutants (Atg3^{P21A} and hAtg3^{L23T}) fold to an α-helix conformation in the presence of liposomes (Fig. S4a and S4b).

We agree with the reviewer and have provided critical experimental data on membrane binding (Fig. S5), thioester formation (Fig. S3b), and *in vitro* conjugation assays (Fig. 3 and Fig. S3a) for all constructs. We employed the liposome co-sedimentation assay to directly examine the membrane binding of hAtg3 and its variants, as suggested by the second reviewer.

As an additional control, we designed a mutant, hAtg3^{V8D_V15K}, that we predict would not be able to interact with the membrane. As expected, the NT^{V8D_V15K} peptide is unfolded in the presence liposomes (Fig. S4c), and the hAtg3^{V8D_V15K} protein displays minimal levels of liposome association, which is similar to a hAtg3 construct without the first 25 residues (Fig. S5). Although it can form a normal covalent thioester intermediate with LC3B (Fig. S3b), the hAtg3^{V8D_V15K} protein does not catalyze the LC3B-PE conjugation.

2) In vivo rescues – Figure 4 needs to have replicates as independent lentiviral transductions. As there is some lipidation in the proline-mutant, the degree of reduced activity could reflect the mutation itself (as authors suggest) or a variance in autophagy activity due to apparently higher expression of this mutant. Several proteins in the lipidation cascade have been shown to inhibit activity in cells when overexpressed (or underexpressed) significantly. Furthermore, could the authors comment on the "flux" they observe? There is no apparent increase in total LC3-II following Baf except under starvation, and even then it does not appear to accumulate beyond what was already present in the unstressed no-Baf condition. These values should be quantified from replicates and included in the figure. Alternatively, another flux substrate such as p62 could be used.

Author reply: We thank the reviewer for the insightful comments. We have repeated the lentiviral transduction combined with cell sorting to obtain comparable expression levels between wildtype and mutant hAtg3 proteins and performed immunoblotting to determine LC3B lipidation and autophagic flux with 6 replicates (Fig. 4a-c). Consistent with the *in vitro* data shown in Fig. 3, when compared to hAtg3, hAtg3^{P21A} only partially rescued LC3B lipidation and hAtg3^{L23T} nearly completely failed to do so in Atg3$^{-/-}$ MEFs (Fig. 4a and b). Although there was no statistical difference in the basal autophagic flux, nutrient starvation induced a significantly high level of LC3-II lysosomal turnover in Atg3$^{-/-}$ cells expressing hAtg3 compared to the empty vector, hAtg3^{L23T} or hAtg3^{P21A} (Fig. 4c). Unfortunately, we did not observe significant differences in p62 protein levels between wildtype and mutant hAtg3 transfectants. Although the underlying mechanisms are not known, we speculate that Atg3$^{-/-}$ MEFs are adapted to low autophagy activity (e.g. low basal autophagic flux and lysosomal turnover of p62), which cannot be rescued by transient or short-term expression of hAtg3. Nevertheless, the major goal of this study is to better understand the role of Atg3 in the LC3-PE conjugation reaction, and our data strongly support that the N-terminal conserved region of hAtg3 plays an essential role in the LC3 lipidation both *in vitro* and *in vivo*.

3*) DATA quality problems: In Figure 3, the top panel in both 3a and 3b appears to be an identical gel. Were all of these experiments conducted on the same day from the same samples? As above, more information on replicates is necessary. In Figure S7, the gel for p21A-0.25Q appears to have the last lane simply pasted on. If this is data from another gel (or from a separate region on the same gel), this piece should be shown as actually separate rather than connected to other samples*.

<u>Author reply:</u> A new Fig. 3 has been prepared to address reviewer concerns, and the full gel images are available as a supplemental figure (Fig. S3a). Fig. S7 (Fig. S11 in the revision) has also been reworked (it was from a separate gel as noted), and full gel images have been included for all experiments.

4) Why do bicelles work when other membrane mimics do not? The authors write that "The observation that hAtg3 is more active in larger bicelles (Fig. S7d) further supports the notion that the protein interacts with the planar surfaces instead of the edges of bicelles that have a curvature radius of approximately 5 nm." This explanation is not obvious to me, as the larger bicelles will also have more curved surface area. If the binding is actually via the curved edge, would this impact the way the authors interpret their story, as a 5 nm radius of curvature is probably not representative of the membranes in a cellular environment?

<u>Author reply:</u> Disc-like bicelles have long chain lipids at the planar surface (DMPC, DMPG, and LPE used in this study), while short chain lipids at the edge (DHPC used in this study). Larger bicells, in fact, proportionally have more planar, but less curved surface than smaller ones. Consequently, if hAtg3 interacts with the curved surface, it should be less active in larger bicelles at the same amount of total lipids. This is in contrast to our experimental observations. Therefore, we suggest that hAtg3 presumably interacts with the planar surface of bicelles, and have revised the text to further clarify this argument.

5) Impact of the deletion mutant (delta 90-190) – The authors are able to make an impressively large deletion in ATG3 without massively impacting its activity, and thus are able to use this deletion for subsequent structure analysis. However, in measuring the activity, they speculate that the reduced lipidation driven by this mutant reflects a reduced rate of ATG3-LC3 conjugate formation due to loss of ATG7 binding. That would suggest this first coupling event is rate-limiting, but the data in SFig4 b and c are hard to square with that interpretation. As in other places, it would be helpful if replicates were shown. Is SFig4b representative, in which case the impact on lipidation is fairly mild, however, in Sfig4C they observe almost no ATG3-LC3 conjugate formation suggesting the enzyme is nearly dead. I wonder if an alternative interpretation is that loss of the loop destabilizes the ATG3-LC3 conjugate and thus it does not survive the sample preparation ahead of running the gel.

<u>Author reply:</u> To probe whether LC3B-hAtg3$^{\Delta 90\text{-}190}$ formation is impaired by the deletion of Atg7 interacting site of hAtg3, we have examined the intermediate formation, the stability of the thioester intermediate in aqueous solution, and LC3-PE conjugation with an increased amount of mouse Atg7 (mAtg7) (Fig. S7b, c and d). The data support that the Atg3-Atg7 interaction is at least one of the rate-limiting steps for the deletion mutant (hAtg3$^{\Delta 90\text{-}190}$), as the reviewer suggested.

6) In vitro conjugation in methods – "The in vitro conjugation was conducted similarly to previous descriptions87,142." There are only 34 references in this paper, please double-check the references in the methods section.

Author reply: We have corrected this error, thank you.

7) Suggestions for clarity -- The nomenclature "NTconv" intended to be short for conserved N-terminus might be clearer as NTcon or NTcons. Note on pg 10, "Fig. S6d" should actually read "Fig. S7d".

Author reply: We agree that NT^{Cons} is more intuitive and have changed the nomenclature throughout. As we have added more supplemental data, all figures have been changed and updated, but we thank the reviewer for catching this error.

Reviewer #2 (Remarks to the Author):

Autophagy is a membrane traffic pathway, in which different cellular materials are sequestered within autophagosomes and transported to lysosomes for degradation. Ubiquitin-like Atg8-family proteins (LC3s and GABARAPs in mammals) are anchored to nascent autophagosomal membranes via their lipidation (conjugation to phosphatidylethanolamine (PE)), and play multiple roles, including autophagosome formation and cargo recognition. Lipidation of Atg8-family proteins is mediated by the ubiquitin-like conjugation reaction, in which Atg3 serves as an E2 enzyme that conjugate the proteins to PE. Previous studies have revealed that the N-terminal region of Atg3 contains an amphipathic helix (AH), which is responsible for membrane binding of this enzyme and thus is essential for its conjugase activity. In this study, Tyndall et al. found that the N-terminal region of Atg3 additionally contains a short conserved segment C-terminal to the AH. This segment was proven to be required for LC3 lipidation but seemed not to be involved in membrane binding of Atg3. NMR analyses suggested that Pro21 in this segment is required for conformational changes in the catalytic core domain of Atg3 that are induced by its membrane (bicelle) binding. Taken together, the authors proposed a model that this conserved segment acts to transmit a signal of membrane binding in the AH to the core domain to stimulate Atg3 conjugase activity. However, several major issues remain to be addressed to allow the authors to draw this conclusion.

Major comments:
(1) The authors should perform the experiments shown in Figs. 4, 6, S3, and S7, and additional experiments requested below for the L23T mutant to know the role for this mammal specific residue.

Author reply: We thank reviewer for this important and insightful remark. We have performed more experiments on L23T and the data are presented in Fig. 4S (peptide folding), Fig. S5 (membrane binding), Fig. S3b (thioester intermediate formation), Fig. 3 and Fig. S3 (in vitro conjugation assay), and Fig. 4 (in vivo cellular assay). These data have further substantiated our conclusion that the NT^{Cons} of hAtg3 is required for LC3-PE conjugation.

The $hAtg3^{L23T}$ construct shows moderate conjugation activity in bicelles (Fig. S11), in contrast to its almost total inactivity in the *in vitro* conjugation assay with liposomes and the *in vivo* cellular assay. This result indicates that bicelles are not a valid model for a structural characterization of the membrane-bound $hAtg3^{L23T}$ using high-resolution NMR. In the future, a structural comparison of membrane-bound structures of hAtg3 and $hAtg3^{L23T}$ in liposomes may be pursued with solid-state NMR or other techniques.

(2) Membrane binding assay based on changes in tryptophan fluorescence should include a

previously-described AH mutant defective in membrane binding to show that the changes were indeed resulted from the membrane binding of Atg3 in the AH. In addition, it would be better if more direct methods such as liposome flotation assay will be performed. CD spectrometry should also be performed for peptides containing the P21A and L23T mutations to confirm that the membrane association property of the mutant proteins was not affected by these mutations.

Author reply: Thank you for the suggestion to use a more direct method of measuring Atg3-liposome association. Membrane binding for all our constructs have been examined using the liposome co-sedimentation assay (Fig. S5). CD spectra for peptides containing the P21A and L23T mutations in the absence and presence of liposomes are presents in Fig. S4.

(3) Confirming thioester intermediate formation of the Atg3 mutants is critical and therefore should be presented in the manuscript.

Author reply: We agree with the reviewer's remark and have included the data in Fig. S3b.

(4) Since in vivo experiments are not clear enough, the quantification will be required. Immunoblot of p62 will also be helpful to evaluate autophagic flux.

Author reply: Please see response to Reviewer #1, comment (2).

(5) The authors should perform CD spectrometry and membrane binding assay using bicelles for wild-type and mutant peptides and proteins to show that the results of these analyses using liposomes can be discussed along with structural information obtained by NMR, in which bicelles were used.

Author reply: While we agree that CD spectrometry using bicelles would be helpful for a comparison to the NMR data, bicelles are not suitable for CD experiments since they produce a very strong CD signal. In addition, bicelles are not suitable for the co-sedimentation assay. However, we were able to use NMR $^{13}C_{\alpha}$ secondary chemical sifts to confirm the helicity of the NT of hAtg3 when bound to bicelles (Fig. S13).

(6) The authors should clearly state whether the P21A mutant did not cause a conformational change upon the addition of bicelles or caused a conformational change different from the wild-type protein.

Author reply: We have amended the text to clearly state that while hAtg3$^{\Delta 90\text{-}190_P21A}$ undergoes a conformational shift upon the addition of bicelles, the structure of membrane-bound hAtg3$^{\Delta 90\text{-}190_P21A}$ differs from the structure of membrane-bound hAtg3$^{\Delta 90\text{-}190}$.

(7) In the present manuscript, there is no evidence supporting the model that the conserved segment works downstream of membrane binding in the AH to cause conformational changes in the core domain to increase Atg3 conjugase activity. The authors have to show that a mutation in the AH that impairs the membrane binding of Atg3 cancels the conformational changes caused by bicelles as with the case for the P21A mutation.

Author reply: To address this question, we designed a double mutant, hAtg3^{V8D_V15K}, that does not interact with liposomes. As described above in response to reviewer #1, the NT^{V8D_V15K} peptide is unfolded in the presence of liposomes (Fig. S4c) and the hAtg3^{V8D_V15K} protein displays minimal liposome association (Fig. S5). In the NMR spectra of the hAtg3$^{\Delta 90\text{-}190_V8D_V15K}$ in the absence and presence of bicelles (Fig. S14b), there are only small perturbations,

indicating that the membrane binding of AH is required to induce conformation rearrangements in hAtg3.

Minor comments:
(1) Page 3, line 3: The phagophore is a cup-shaped "single" membrane vesicle.

Author reply: Corrected.

(2) Page 3, line 12: Atg4 removes not the last residue but several residues from the C-terminal region of LC3.

Author reply: Clarified, and thank you for the suggestion.

(3) In some parts in the text, the authors use the words "temporally" or "spatio-temporal", but the focus of the present study is not related to the temporal regulation of LC3 lipidation or autophagosome biogenesis.

Author reply: We have substituted them with the word of “spatial”, as we were examining membrane geometry driven behavior.

(4) The relationship between human and yeast proteins should be described as "homologs or orthologs" but not "isoforms".

Author reply: Corrected.

(5) Page 10, line 12: "Fig. S6d" should be "Fig. S7d".

Author reply: We have updated all figure references.

(6) It may be better if the positions of Y220, S237, E248, and H252 are also shown in Fig. 5c.

Author reply: We have colored these residues in Fig. 5c.

Reviewer #3 (Remarks to the Author):

The manuscript reports the identification of a - hitherto overlooked - conserved sequence motif in the N-terminal region of the autophagy-related protein Atg3, which plays an essential role in autophagosome formation. By investigating several yeast-human chimeric constructs via in vitro and in vivo assays the authors show that this motif is required for efficient conjugation of LC3 family proteins to phospholipids at the tip of the autophagic membrane. An extensive set of NMR spectroscopic experiments suggests that this motif couples the coil-to-helix transition of the N-terminal membrane curvature sensor to a conformational change near the active center in a manner reminiscent of allosteric regulation.

The paper is based on an extensive body of convincing experimental data. The results are of sufficient novelty and of considerable biological interest, and in the discussion the authors point out (convincingly again) that the study opens up follow-up studies geared at understanding the exact mechanism of this conformational switch. Unfortunately, the level of detail given in the present manuscript does not always live up to the standard of allowing an expert reader to fully

understand and reproduce the experiments. I would therefore recommend publication of this manuscript in Nature Communications after major revisions, as described below.

While this interesting manuscript is written in a very readable style overall and has the potential to become a real pleasure to read in a revised version, I found the presentation of the sequence alignments a little difficult to digest. It is split between the introduction, results section, Figs. 1, 2, and S1. The font size in the figures is rather small and hard to read. The color codes used in the figures are different from figure to figure, largely unexplained, and not particularly intuitive. The order of species in the sequence alignments also appears pretty random to me. All the necessary information can be found somewhere in these sections, but I think the presentation of this important part of the manuscript could really benefit from a careful make-over towards a clearer and more unified presentation.

Author reply: We apologized for the confusions, and have taken the reviewer's suggestions to heart and colored the residue so that they are consistent between these two figures and explicitly detailed the color mappings in the figure legends. We divided the discussion of the NTs between sequence comparison (Fig. 1) and predicted helical structure comparison (Fig. 2). The sequence order in Fig. 1 has human and yeast bordering for ease of comparison, and after that, we grouped animals and fungi separately, which may have led to the random grouping appearance.

Maybe my biggest concern is the general lack of detail provided, especially in the figure legends. There are more unexplained color codes, abbreviations, symbols, units etc. in this manuscript – especially in the figure legends – than I can list here. In the case of Fig. 4, the legend is so sparse that I could not even understand the figure any more. The NMR spectra are presented without any details, not even the magnetic field strength used, even though it is important for the peak position in TROSY spectra. And the structural models and overlays are small and unlabeled, not even the N- and C-termini are marked, which makes the orientation very cumbersome. And there are several sentences in the manuscript with a missing or unclear (literature or figure) reference.

Author reply: We have provided more detail in the figure legends. Per the reviewer's remark, NMR data acquisition parameters have also been included in Methods and the C and N terminals of the structural models have been labeled. Finally we have added references in areas where it was unclear, and have clarified and updated the figure reference.

The authors present sequence-specific NMR resonance assignments and even use these chemical shifts for homology modeling using PONOMA, but they have not made the underlying chemical shifts publicly available. It is standard practice in the community and (as far as I know) a requirement of the editorial policy of Nature journals to deposit chemical shift assignments with the BMRB before publication. It might also be worthwhile to make the PONOMA results available for download, but this is optional.

Author reply: NMR resonance assignments for hAtg$^{\Delta 90\text{-}190}$ in aqueous solution and in bicelles, hAtg3$^{\Delta 90\text{-}190_P21A}$ in bicelles, and hAtg3$^{\Delta 1\text{-}25}$ in aqueous solution have been deposited with the BMRB with accession numbers #50479, #50480, #50481, and #50470, respectively. The PONOMA modeling results of hAtg3$^{\Delta 1\text{-}25}$ will be available upon request.

Individual comments:
** Abstract: "structurally distinct" is not entirely clear here.*

Author reply: We have rephrased the sentence to clarify, thanks.

** In humans, Atg8 is not a single protein but a family of at least 6 proteins divided into two sub-families (GABARAP-like and LC3A/B/C). The expression "LC3" used throughout this manuscript is therefore highly confusing. Please clarify exactly which protein(s) each instance of "LC3" refers to. From what I understand, the authors mostly use LC3B, which raises the question whether the results could be different for other members of the LC3/GABARAP family. Please discuss.*

Author reply: We thank the reviewer for the insightful comments. We have updated the manuscript to reflect the specific homolog we used, LC3B, and added a discussion of GABARAP and other homologs as well. Although the LC3/GABARAP superfamily members share structurally similar functional domains, recent studies have revealed that each of the members may have a distinct role in the process of autophagy (Nguyen, T. et al. (2016), J. Cell. Biol. 215: 857-874; Grunwald, D. S. et al. (2020), Autophagy, 16: 600-614). Thus, the N-terminal region of Atg3 might influence the lipidation reaction among the family members differently. We agree with the reviewer that further exploring this area would be interesting for future studies.

** "we synthesized two peptides that correspond to the NT regions of hAtg3 and yAtg3 (Fig. 1a)" – I cannot see these constructs in Fig. 1a.*

Author reply: While the constructs are not explicitly listed, they have the same sequence as listed for human and yeast NT in Fig.1a. We have provided the exact sequences for these two peptides in Methods, and clarified the text to reflect that.

** Not just the figure legend to Fig. 4 is a little sparse, so is the explanation in the main text. I do not think the readers should be forced to read ref. 20 to be able to comprehend the present manuscript.*

Author reply: We have updated Fig. 4 extensively with additional *in vivo* data and revised the text accordingly.

** "their resonances are not observed as a result of intermediate exchange" - Is there any hard evidence that this can be attributed to chemical exchange, or is this just a (plausible) hypothesis? Please clarify.*

Author reply: This is a plausible hypothesis. We have rephrased this sentence to "their resonances are not observed, most likely as a result of intermediate exchange".

** "show good convergence […] (Fig. S6)." - I cannot see ten structural models in Fig. S6 and therefore no RMSD and no convergence.*

Author reply: We have provided an overlay of ten lowest-energy structural models of hAtg3$^{\Delta 1\text{-}25}$ in Fig. S10a.

** "comparable to that in liposomes (Fig. S6d)" – could this be a reference to Fig. S7d?*

Author reply: We have updated the text and figure.

** "Atg3 is more active in larger bicelles (Fig. S7d)" – I do not find this straightforward to see in Fig. S7d.*

Author reply: We have provided a better, larger image (Fig. 11c) to show that at 240 min, close to 90% of the LC3B-PE is conjugated in larger bicelles, while in smaller ones ~55% LC3B-PE conjugation occurred.

** From what I understand (difficult to say due to the lack of detail in the methods section), the chemical shifts of hAtg3 bound to bicelles had to be determined from triple-resonance experiments, which suggests to me that 13C chemical shifts are available as well. 13C chemical shifts are highly sensitive to any changes in secondary structure. Are there any detectable changes in regular secondary structure upon membrane binding, or are the "structural rearrangements" more subtle?*

Author reply: There are large changes in ^{13}C chemical shifts for the NT residues since they transition from random coil to alpha helix. On the other hand, we do not know whether there are perturbations in ^{13}C shifts for residues 260 to 269 around the active site, C264, since they are not assigned in aqueous solution. In addition, while changes in ^{13}C shifts for most other residues are small, they are not indicative of tertiary structural rearrangements, since $^{13}C_{\alpha}$ and $^{13}C_{\beta}$ shifts are mainly sensitive to changes in secondary structure.

** "albeit with decreased Atg12-Atg5 conjugation" – this sentence was not clear to me.*

Author reply: We have rephased the sentence.

** "similar to the configuration seen in the active site" (article missing)*

Author reply: We have added references.

** Legend to Fig. 3: Not sure "respectively" is used correctly here.*

Author reply: A better phrase was substituted for respectively.

** "was exchanged to a thrombin cleavage buffer" – how was the buffer exchange performed?*

Author reply: We have rephrased this sentence.

** "BME" – beta-mercaptoethanol?*

Author reply: We have updated the first occurrence to include the full name.

** I don't think there are any references numbered 87 and 142.*

Author reply: We have updated these references.

** "reaction buffer (50 mM HEPES, 150 mM NaCl" (typo)*

Author reply: Corrected, thanks.

** "Gels were destained" - or is there such a thing as a detention center for gels? ;-)*

Author reply: Corrected.

** "with a 0.5 mm bandpass" – mm or nm?*

Author reply: This whole section has been removed and replaced with the liposome sedimentation assay.

** "hAtg3yMin" – which construct is this?*

Author reply: The name was from an earlier draft. We have updated it to hAtg3^{NTCons}.

** The NMR methods section is far, far, far too sparse on details and references.*

Author reply: We have added more detail to the NMR methods, thank you for the feedback.

** "For the stability of samples with 12% bicelles the pH was kept at 7.5." – unclear*

Author reply: We have expanded the text to indicate that at pH 6.5, the bicelle sample was not stable for long-term NMR experiments.

** Fig. 5a is a bit on the small side.*

Author reply: We have enlarged NMR figures to increase legibility.

** Fig. 6c: "aqueous" (typo)*

Author reply: Corrected.

** Fig. S2: "require the presence of negatively charged lipids (PGs or PIs)" – I cannot see any control without PGs or PIs? Also, exactly which lipids were used?*

Author reply: We have added Fig. S2c to show that NT folding requires the presence of negatively charged lipids.

** Fig. S3: Why does the Trp fluorescence decrease upon liposome binding? Naively, I would have expected it to become more protected from water and hence to increase...*

Author reply: We believe it is due to the fact that the NT only has one of the 5 tryptophan in the protein, and the fluorescence changes from others may impact the overall observed fluorescence. This figure is not included in the revision, since we have chosen to use the liposome co-sedimentation assay to directly examine the membrane binding of hAtg3 and its variants, as suggested by the second reviewer.

** Fig. S4b: The figure resolution looks a bit pixelated. Also, "Atg7m" is not explained, I think.*

Author reply: We have updated with higher resolution images (Fig. S7 in revision). In the figure legend we clarify that mAtg7 is mouse Atg7.

** Fig. S7c is definitely too small.*

Author reply: We have enlarged this figure (Fig. S12 in revision).

** Fig. S8: How was the secondary chemical shift calculated (i. e., what is Ciso)? (Reference necessary.) Upon closer inspection, there is a systematic downfield shift of about 1.5 ppm even in buffer, which exceeds the CSI cutoff of +0.8 ppm for helical conformation (Wishart & Sykes, Meth. Enzymol. 239, 363-392), suggesting that there is already significant helical propensity. And the downfield shift of 4..5 ppm with bicelles is larger than expected ("As a general rule, 13Ca shifts experience a downfield shift of about 2.5 ppm in helices", Wishart & Case, Meth. Enzymol. 338, 3-34). Please double-check the referencing. Was this a deuterated sample? If so, did you correct for isotope effects?*

<u>Author reply:</u> Thanks for the feedback. We have fixed a bug in one of our scripts used for data analysis, double-checked the referencing, and corrected isotope effects. A new plot is shown in Fig. S13.

<u>Reviewer #4 (Remarks to the Author):</u>

The manuscript by Tyndall et al. entitled «An N-terminal conserved region in human Atg3 couples membrane curvature sensitivity to conjugase activity during autophagy» shed a light on a very interesting and important topic – how the activity of autophagy E2 ligase Atg3 (expressed in conjugation of human Atg8 homologs LC3/GABARAP proteins to phosphatidylethanolamine) correlates with the specific membrane curvature at the edges of growing autophagocome. N-terminal evolutionary conserved region of Atg3 proteins was recently identified to form an amphipathic helix (AH), which senses membrane curvature and couples Atg3 conjugation activity to the growing phagophore. However, the mechanism of this correlation remained unknown. The authors made a big step toward to understanding of the relationship between functional and structural interplay within the Atg3 family proteins. They analyzed the N-terminal region (NT) of Atg3 proteins (residues 1-26 in human Atg3) and found that residues 3-19 are involved in the AH formation. Residues 1-16 are rather variable (NTVar), while residues 17-26 are highly conserved NTConv). The authors unambiguously demonstrated that NT adopts a helical conformation exclusively in presence of the small-size liposomes, being unstructured in aqueous solution or in presence of larger size liposomes. By in-vitro conjugation assays and by ability of several Atg3 chimeric constructs and mutants to rescue LC3 lipidation in Atg3-/- mouse embryonic fibroblasts (in-vivo), authors demonstrated that the Atg3 activity lost is predominantly due to alteration of the NTConv. In order to understand molecular basis of the observed functional significance of NTConv, they undertook a series of NMR experiments to visualize chemical shift perturbations (SCP) in presence of curved membrane-mimicking bicelles (here I should mention that the NMR work presented here - including the Atg3 constructs optimization, screening of membrane-mimicking components and assignment of resonances in the media - is extremely elegant and informative). Results of these experiments indicate that in presence of the corresponding bicelles i) Atg3 residues 3-19 form a helical structure; ii) significant CSP are observed at the C-terminally located catalytic core of Atg3, indicating structural rearrangements of the core. Comparison of the CSP for Atg3 and its P21A mutant (the mutant with almost complete abolished LC3 lipidation activity in-vitro and in-vivo) reveal that the P21A mutation affects both NT and catalytic core structures, disrupting productive interaction between both and inactivating Atg3.

In summary, this study provides new and important insights on the functional correlation between the N-terminal membrane-curvature sensing part of Atg3 and its C-terminal catalytically active part. Experimental designs are solid, and the results are clear and sufficient to draw the conclusions the authors made in the manuscript. Almost all necessary controls were

performed and obtained results were consistently interpreted. Therefore, I strongly recommend this research paper for publication in "Nature Communications" after minor revision.

Author reply: We highly appreciate the feedbacks and encouraging remarks.

I have just a few concerns:
The hAtg3 optimized construct used for NMR experiments (hAtg3Δ90-190) was approved to be able to lipidate LC3 in-vitro (Fig S4b) but its inactive mutant - hAtg3Δ90-190,P21A - was not tested in-vitro, gaining the lack of this negative control.

Author reply: We have performed the *in vitro* conjugation assay for $hAtg3^{\Delta 90\text{-}190\_P21A}$, and the result is shown in Fig S7d.

Additionally, the both deletion constructs were not tested in-vivo (LC3 lipidation level and autophagy flux for hAtg3Δ90-190 and hAtg3Δ90-190,P21A in the Atg3-/- MEFs, the rescuing experiments). I believe that these experiments are necessary as kind of positive controls which bring together the structural, biochemical and cellular parts of the story. In that case the results and conclusions made by authors will be more convincing.

Author reply: We did not test $hAtg3^{\Delta 90\text{-}190}$ and $hAtg3^{\Delta 90\text{-}190\_P21A}$ in *in vivo* cellular assay because a previous study has shown that disrupting the Atg7 interacting site of yAtg3 abolishes Atg8 (LC3's counterpart in yeast) lipidations *in vivo* (Yamada, Y. et al. (2007), J. Bio. Chem. 282: 8036-8043). We have added this reference to the manuscript.

Minor points:
1. At Figure 1b the sequences might be centered on the EYLTP sequence for better understanding.

Author reply: Thank you for an excellent suggestion, and we have done so.

2. Figure S7c is too small to be clearly seen, authors should increase either size of the NMR plot or image resolution.

Author reply: We have made this panel its own figure (Fig. S12), and increased the resolution.

REVIEWERS' COMMENTS

Reviewer #1 (Remarks to the Author):

In this revised manuscript by Tyndall et al, my major concerns have each been addressed. In particular, the authors provide replicates and statistical analysis to back up most of their original claims, and have greatly expanded the supplemental material to present a rigorous study. In direct response to my questions, they have much more firmly established that the deletion mutant they are using is specifically impacting ATG7 interactions (Fig. S7). On the whole, I expect this paper will be of interest to the broader autophagy community.

The only minor remaining concern I have is with their interpretations of the L23T mutant. This amino acid is strongly conserved in mammals and the authors suppose it marks a key linker residue in the connection of NT membrane binding and active site reorganization. The authors are not able to explore this reorganization at the NMR level because this mutant surprisingly retains some activity on bicelles. However, their membrane-binding activity suggests a simpler answer – the partial loss of activity by this mutant in some assays may be directly correlated to a partial loss of membrane binding, and as such, I am not sure this is a great example of a putative linker transduction region.

Line 152 – "The results of CD and liposome co-sedimentation assays21, 22 allow us to rule out the 153 possibility that the effects of these substitutions are due to a defect in protein-membrane 154 interactions. Both hAtg3P21A and hAtgL23T interact with the liposomes in an analogous manner to 155 wildtype protein (Fig. S4a and b, S5)." – both figure S4 and S5 look to me to be consistent with an interpretation that membrane binding is reduced, having lost approximately half of the NT-dependent pelleting and something similar in CD, for the L23T mutant. As the authors also observe lipidation on bicelles for this mutant, collectively, I am not sure they can draw any conclusions about the role of L23 in transducing membrane binding information to conformational changes.

Reviewer #2 (Remarks to the Author):

I found the authors have satisfactorily addressed all the issues I raised for the initial manuscript.

Reviewer #3 (Remarks to the Author):

The manuscript reports the identification of a - hitherto overlooked - conserved sequence motif in the N-terminal region of the autophagy-related protein Atg3, which plays an essential role in autophagosome formation. By investigating several yeast-human chimeric constructs via in vitro and in vivo assays the authors show that this motif is required for efficient conjugation of LC3B to phospholipids at the tip of the autophagic membrane. An extensive set of NMR spectroscopic experiments suggests that this motif couples the coil-to-helix transition of the N-terminal membrane curvature sensor to a conformational change near the active center in a manner reminiscent of allosteric regulation.

The paper is based on an extensive body of convincing experimental data. The results are of sufficient novelty and of considerable biological interest, and in the discussion the authors point out (convincingly again) that the study opens up follow-up studies geared at understanding the exact mechanism of this conformational switch. In the revised version the results are presented in a greatly improved and very readable fashion and the authors have addressed the points raised by the reviewers to my satisfaction. I would therefore recommend publication of this manuscript in Nature Communications.

I only have a few minor points to add:

* Do the authors have any explanation for the behavior of the L23T mutant (lines 235 following)?
* On a first glance, it is a bit surprising that the Atg3 curvature sensor binds to (packing defects in?) the planar face of the bicelles rather than to the curved edges, as the comparison of the two bicelles with different q values suggests. Do I understand that correctly that the authors believe the curvature radius of the edges of 5 nm (line 231) to be too small, or what is the rationale? Maybe this can be clarified in the manuscript.
* In Fig. S13, I can only see support for 13Calpha changes for residues 3 to 18, but not 19?
* I am still not entirely happy with Fig. S13, even though the numbers look much more reasonable now. Please specify exactly (the emphasis is on exactly here) which random coil chemical shifts were used – we need a peer-reviewed reference here, not just some unpublished manual that Bruker ships with its spectrometers. And please specify exactly how the deuterium isotope effects were corrected for (again, a peer-reviewed reference would come handy). This information is absolutely required to be able to replicate the results.
* I would highly recommend references for the TROSY triple-resonance pulse sequences used by the authors (there are, in fact, different TROSY schemes so this information is not entirely expendable).
* I would also recommend specifying the temperature (25°C, I think) and maybe the pH in the legends to all TROSY spectra. It just makes it so much easier for the reader, even if this information can be found somewhere in the methods section.
* I have never seen the qualifier "reasonable" in the data availability statement (line 416) before, but I will leave that up to journal policy.
* There are still a few grammatical mistakes in the manuscript, I noticed a few missing articles ("despite the large size", l. 242/243; "the beginning of the last alpha-helix", l. 261;"seen in the active site", l. 299; "interaction with the membrane", legend to Fig. S14), the sentence on l. 268 looks weird to me (how about: "a mutant that is unable to bind the membrane and has no"), "intrinsically disordered protein (l. 326), "small amount of degradation" (l. 380).

Response to the Reviewers

We would like to thank the reviewers again for their stimulating and constructive comments, and have carefully revised the manuscript following their suggestions.

Reviewer #1 (Remarks to the Author):

In this revised manuscript by Tyndall et al, my major concerns have each been addressed. In particular, the authors provide replicates and statistical analysis to back up most of their original claims, and have greatly expanded the supplemental material to present a rigorous study. In direct response to my questions, they have much more firmly established that the deletion mutant they are using is specifically impacting ATG7 interactions (Fig. S7). On the whole, I expect this paper will be of interest to the broader autophagy community.

The only minor remaining concern I have is with their interpretations of the L23T mutant. This amino acid is strongly conserved in mammals and the authors suppose it marks a key linker residue in the connection of NT membrane binding and active site reorganization. The authors are not able to explore this reorganization at the NMR level because this mutant surprisingly retains some activity on bicelles. However, their membrane-binding activity suggests a simpler answer – the partial loss of activity by this mutant in some assays may be directly correlated to a partial loss of membrane binding, and as such, I am not sure this is a great example of a putative linker transduction region.

Line 152 – "The results of CD and liposome co-sedimentation assays21, 22 allow us to rule out the possibility that the effects of these substitutions are due to a defect in protein-membrane interactions. Both hAtg3P21A and hAtgL23T interact with the liposomes in an analogous manner to wildtype protein (Fig. S4a and b, S5)." – both figure S4 and S5 look to me to be consistent with an interpretation that membrane binding is reduced, having lost approximately half of the NT-dependent pelleting and something similar in CD, for the L23T mutant. As the authors also observe lipidation on bicelles for this mutant, collectively, I am not sure they can draw any conclusions about the role of L23 in transducing membrane binding information to conformational changes.

Author reply: As the reviewer has correctly stated, NT helicity and membrane association are lower for L23T compared to WT (Supplementary Fig. 4, 5). In the liposome sedimentation assay, L23T shows ~60% of the binding of the WT protein (Supplementary Fig. 5). However, the moderate decrease in liposome binding is not sufficient to explain the near-complete inactivation of L23T in *in vitro* conjugation assay with liposomes and *in vivo* cellular assay (Fig. 3, 4). In addition, the decreased helicity seen in the CD results could be partially due to the decreased binding of the mutant to lipopsomes besides a structural difference between the folded L23T and the WT NT. These data suggest that L23 may play a role in both signal transduction and membrane binding. We have revised the text to reflect this. On the other hand, the observed lipidation of L23T mutant in bicelles may reflect limitations of this membrane model.

Reviewer #2 (Remarks to the Author):

I found the authors have satisfactorily addressed all the issues I raised for the initial manuscript.

Author reply: Thank you again for your valuable feedbacks.

Reviewer #3 (Remarks to the Author):

The manuscript reports the identification of a - hitherto overlooked - conserved sequence motif in the N-terminal region of the autophagy-related protein Atg3, which plays an essential role in autophagosome formation. By investigating several yeast-human chimeric constructs via in vitro and in vivo assays the authors show that this motif is required for efficient conjugation of LC3B to phospholipids at the tip of the autophagic membrane. An extensive set of NMR spectroscopic experiments suggests that this motif couples the coil-to-helix transition of the N-terminal membrane curvature sensor to a conformational change near the active center in a manner reminiscent of allosteric regulation.

The paper is based on an extensive body of convincing experimental data. The results are of sufficient novelty and of considerable biological interest, and in the discussion the authors point out (convincingly again) that the study opens up follow-up studies geared at understanding the exact mechanism of this conformational switch. In the revised version the results are presented in a greatly improved and very readable fashion and the authors have addressed the points raised by the reviewers to my satisfaction. I would therefore recommend publication of this manuscript in Nature Communications.

I only have a few minor points to add:
** Do the authors have any explanation for the behavior of the L23T mutant (lines 235 following)?*

Author response: Currently we do not have a structural model to explain the behavior of the L23T mutant since bicelles is not a valid membrane model for its structural study using high-resolution NMR. A solid-state NMR study, using liposomes, may be able to provide an answer to this question in the future.

** On a first glance, it is a bit surprising that the Atg3 curvature sensor binds to (packing defects in?) the planar face of the bicelles rather than to the curved edges, as the comparison of the two bicelles with different q values suggests. Do I understand that correctly that the authors believe the curvature radius of the edges of 5 nm (line 231) to be too small, or what is the rationale? Maybe this can be clarified in the manuscript.*

Author response: We do not know if the curvature of the bicelle edges is too high for Atg3 to bind, but a curvature radius of less than 10 nm is not physiologically relevant (Huang et al., (2017), *Proc. Natl. Acad. Sci. USA*, 114: 2910-2915). In addition, bicelles are a dynamic structure and even the planar face is not entirely planar.

** In Fig. S13, I can only see support for 13Calpha changes for residues 3 to 18, but not 19?*

Author response: In the updated Supplementary Fig. 14, $^{13}C_{\alpha}$ secondary chemical shift for residue 19 changes 0.65 ppm.

** I am still not entirely happy with Fig. S13, even though the numbers look much more reasonable now. Please specify exactly (the emphasis is on exactly here) which random*

coil chemical shifts were used – we need a peer-reviewed reference here, not just some unpublished manual that Bruker ships with its spectrometers. And please specify exactly how the deuterium isotope effects were corrected for (again, a peer-reviewed reference would come handy). This information is absolutely required to be able to replicate the results.

Author response: This figure (Supplementary Fig. 14) has been replotted with published random coil shifts. References for random coil shifts and corrections of the deuterium isotope effects were included.

** I would highly recommend references for the TROSY triple-resonance pulse sequences used by the authors (there are, in fact, different TROSY schemes so this information is not entirely expendable).*

Author response: References for these pulse sequences were provided in Supplementary Table 1.

** I would also recommend specifying the temperature (25°C, I think) and maybe the pH in the legends to all TROSY spectra. It just makes it so much easier for the reader, even if this information can be found somewhere in the methods section.*

Author response: This information has been added in the figure legends.

** I have never seen the qualifier "reasonable" in the data availability statement (line 416) before, but I will leave that up to journal policy.*

Author response: We believe that this statement is in consistent with editorial policy, but have edited it.

** There are still a few grammatical mistakes in the manuscript, I noticed a few missing articles ("despite the large size", l. 242/243; "the beginning of the last alpha-helix", l. 261;"seen in the active site", l. 299; "interaction with the membrane", legend to Fig. S14), the sentence on l. 268 looks weird to me (how about: "a mutant that is unable to bind the membrane and has no"), "intrinsically disordered protein (l. 326), "small amount of degradation" (l. 380).*

Author response: We have fixed these typos, thank you.
